# The short- and long-term associations of particulate matter with inflammation and blood coagulation markers: A meta-analysis^☆^

**DOI:** 10.1016/j.envpol.2020.115630

**Published:** 2020-12

**Authors:** Hong Tang, Zilu Cheng, Na Li, Shuyuan Mao, Runxue Ma, Haijun He, Zhiping Niu, Xiaolu Chen, Hao Xiang

**Affiliations:** a Department of Global Health, School of Health Sciences, Wuhan University, 115# Donghu Road, Wuhan, China; b Global Health Institute, Wuhan University, 115# Donghu Road, Wuhan, China; c School of Chemistry, Chemical Engineering and Life Sciences, Wuhan University of Technology, 122# Luoshi Road, Wuhan, China

**Keywords:** Particulate matter, Inflammation, Blood coagulation, Meta-analysis

## Abstract

Inflammation and the coagulation cascade are considered to be the potential mechanisms of ambient particulate matter (PM) exposure-induced adverse cardiovascular events. Tumor necrosis factor-alpha (TNF-α), interleukin-6 (IL-6), interleukin-8 (IL-8), and fibrinogen are arguably the four most commonly assayed markers to reflect the relationships of PM with inflammation and blood coagulation. This review summarized and quantitatively analyzed the existing studies reporting short- and long-term associations of PM_2.5_(PM with an aerodynamic diameter ≤2.5 μm)/PM_10_ (PM with an aerodynamic diameter≤10 μm) with important inflammation and blood coagulation markers (TNF-α, IL-6, IL-8, fibrinogen). We reviewed relevant studies published up to July 2020, using three English databases (PubMed, Web of Science, Embase) and two Chinese databases (Wang-Fang, China National Knowledge Infrastructure). The OHAT tool, with some modification, was applied to evaluate risk of bias. Meta-analyses were conducted with random-effects models for calculating the pooled estimate of markers. To assess the potential effect modifiers and the source of heterogeneity, we conducted subgroup analyses and meta-regression analyses where appropriate. The assessment and correction of publication bias were based on Begg’s and Egger’s test and “trim-and-fill” analysis. We identified 44 eligible studies. For short-term PM exposure, the percent change of a 10 μg/m^3^ PM_2.5_ increase on TNF-α and fibrinogen was 3.51% (95% confidence interval (CI): 1.21%, 5.81%) and 0.54% (95% confidence interval (CI): 0.21%, 0.86%) respectively. We also found a significant short-term association between PM_10_ and fibrinogen (percent change = 0.17%, 95% CI: 0.04%, 0.29%). Overall analysis showed that long-term associations of fibrinogen with PM_2.5_ and PM_10_ were not significant. Subgroup analysis showed that long-term associations of fibrinogen with PM_2.5_ and PM_10_ were significant only found in studies conducted in Asia. Our findings support significant short-term associations of PM with TNF-α and fibrinogen. Future epidemiological studies should address the role long-term PM exposure plays in inflammation and blood coagulation markers level change.

## Highlights

• The first review about associations of PM with TNF-α, IL-6, IL-8, and fibrinogen.
• Short-term PM exposure was significantly associated with TNF-α and fibrinogen.
• Short-term PM exposure was associated with IL-6 in studies conducted in Asia.

## Graphical abstract

**Figure:**
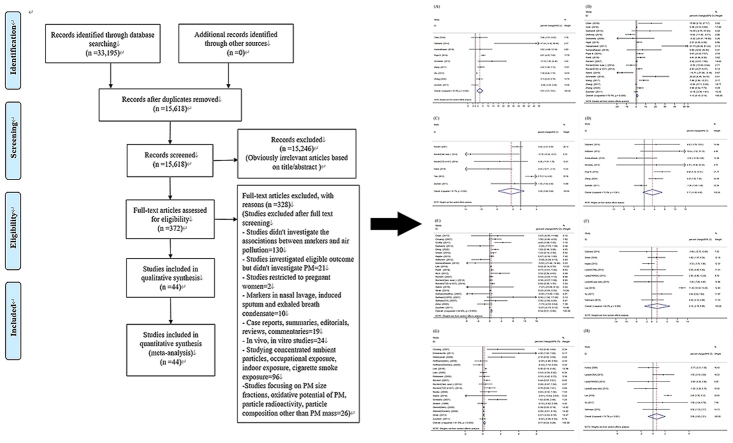

## Teaser

This is the first review about associations of PM with TNF-α, IL-6, IL-8 and fibrinogen. Short-term PM exposure was associated with TNF-α and fibrinogen.

## 1 Introduction

Inflammation and the coagulation cascade are considered as potential mechanisms of ambient particulate matter exposure induced adverse cardiovascular events (Hamanaka and Mutlu, 2018). TNF-α (tumor necrosis factor-α), IL-6 (interleukin-6), IL-8 (interleukin-8), and fibrinogen are arguably the four most commonly assayed markers to reflect the associations of ambient particulate matter with inflammation and blood coagulation (Fang et al., 2012).

There are close links between inflammation and blood coagulation. Inflammation is thought to regulate blood coagulation and activate the fibrinolytic system (Esmon, 2003). For example, acute inflammation can lead to an increase in fibrinogen (Luyendyk et al., 2019). Fibrinogen is a blood coagulation biomarker with proinflammatory effect, which not only play a significant role in platelet aggregation and thrombosis (Kattula et al., 2017), but also increases in response to inflammation (Hoppe, 2014). A study reported that fibrinogen is up-regulated after being stimulated by inflammatory cytokines, such as interleukin 6 (Ridker et al., 2000). Blood coagulation, in turn, play an important role in inflammation. Fibrinogen is one of the most effective contributors to inflammation among all proteins of the coagulation system (Castell et al., 1990). Fibrinogen is considered a potential driver of inflammation-related diseases (sepsis, endotoxemia, encephalomyelitis or multiple sclerosis) (Davalos and Akassoglou, 2012). Studies have shown that fibrinogen can activate inflammation, leading to the release of inflammatory cytokines, such as TNF-α (Jensen et al., 2007). Herein, we focus on four typical biomarkers, which have not only been widely studied in air pollution research to reflect the role of particulate matter in inducing inflammation and blood coagulation, but also related to cardiovascular diseases.

Fibrinogen is regarded as a risk factor and predictor of cardiovascular disease (De Luca et al., 2011; Kunutsor et al., 2016). Studies indicated that fibrinogen was associated with cardiovascular morbidity and mortality (D’Angelo et al., 2006). A meta-analysis reported a significant association of fibrinogen with myocardial infarction (Fibrinogen Studies et al., 2005). In addition, studies also reported that the additional measurement of fibrinogen could help prevent cardiovascular events (Emerging Risk Factors et al., 2012; Maresca et al., 1999). TNF-α, IL-6, and IL-8 are regarded as critical inflammation markers and play a significant role in inflammation (Ghasemi et al., 2011; Mehaffey and Majid, 2017; Unver and McAllister, 2018). Moreover, TNF-α is closely related to atherosclerosis as it contributes to inflammation as well as promoting insulin resistance (Popa et al., 2007). Studies also reported that IL-6 and IL-8 are associated with multiple cardiovascular diseases, such as coronary artery disease, atherosclerosis, sudden cardiac death (Apostolakis et al., 2009; Hussein et al., 2013).

Current epidemiological studies reported inconsistent effects of PM_2.5_ and PM_10_ on the above markers. Among 6589 nonsmoking subjects in South Korea, for short-term PM exposure, Lee et al. reported 0.44% (95%CI: 0.15%, 0.73%) higher fibrinogen levels with 10.4 μg/m^3^ increment of PM_2.5_ and 0.61% (95%CI: 0.33%, 0.90%) higher fibrinogen levels with 20.1 μg/m^3^ increment of PM_10_ (Lee et al., 2018). In healthy college students, for short-term PM exposure, Wang et al. reported the percent change of a 10 μg/m^3^ PM_2.5_ increase on IL-6 and TNF-α was 4.1% (95%CI: 1.2%, 6.9%) and 4.4% (95%CI: 1.7%, 7.0%), respectively (Wang et al., 2018). However, there were studies reported inconsistent findings. A study conducted on general population reported an insignificant short-term association between PM_10_ and fibrinogen (Liao et al., 2005). Among healthy humans, Kumarathasan et al. reported insignificant changes of TNF-α, IL-6, and IL-8 with short-term PM_2.5_ exposure (Kumarathasan et al., 2018).

To date, there has been no meta-analysis to summarize associations of PM (PM_2.5_, PM_10_) with inflammation and blood coagulation markers (TNF-α, IL-6, IL-8, fibrinogen). To fill this gap, this review summarized and quantitatively analyzed the existed studies, which could provide healthcare professionals and researchers with a comprehensive overview of the effect of short-term and long-term exposure to particulate air pollution on TNF, IL-6, IL-8, and fibrinogen.

## 2 Methods

Details of a PRISMA checklist (Moher et al., 2009) were present in the Supplementary material.

### 2.1 Search methods

We searched three English databases (PubMed, Web of Science, Embase) and two Chinese databases (Wang-Fang, China National Knowledge Infrastructure) to identify epidemiological studies that examined the short-term and long-term associations of PM_2.5_/PM_10_ with inflammation and blood coagulation markers up to July 2019. Supplemental Table S1 showed the PECOS statement of all included studies (Morgan et al., 2018). Keywords included (1) “air pollution”, “air pollutants”, “air environmental pollutants”, “environmental air pollutants”, “pollution”, “pollutant∗", “particulate matter”, “particulate air pollutants”, “particulate matters”, “particulate∗", “particle∗", “PM”, “PM_2.5_”, “PM_10_”; (2) “fibrinogen”, “blood coagulation factor I"; (3) “tumor necrosis factor-alpha”, “tumor necrosis factor alpha”, “tumor necrosis factor”, “TNFalpha”, “TNF-alpha”; (4) “Interleukin-6”, “IL-6”, “Interleukin 6”, “IL6”, “Interleukin-8”, “IL-8”, “Interleukin 8”, “IL8”. Also, synonyms of relative markers and particulate matter were searched using Medical Subjects Headings terms. Search strings were summarized in the supplementary material.

### 2.2 Inclusion and exclusion criteria

We evaluated the effects of short-term (for days or weeks) (Lee et al., 2017) and long-term PM exposure (more than six months) (Rodosthenous et al., 2018) on inflammation and blood coagulation markers. The included articles should be epidemiologic studies focusing on the associations of inflammation and blood coagulation markers with PM exposure and reported associations and 95% confidence intervals directly or data could be used to calculate. We excluded *in vivo* studies, *in vitro* studies, case reports, summaries, reviews, editorials, commentaries, and studies that reported inflammation and coagulation markers in nasal lavage, induced sputum and exhaled breath condensate (EBC). Studies restricted to pregnant women (Braithwaite et al., 2019) and focusing on PM size fractions, concentrated ambient particles (CAPs), occupational exposure, indoor exposure, and cigarette smoke exposure were not included.

### 2.3 Study selection

We downloaded all studies identified from five databases into a reference manager (Endnote X8) and removed duplicates. The remaining studies were screened for eligibility by two investigators. First, two investigators screened titles and abstracts to select eligible studies. Then, the remaining studies were reviewed in full texts. Two investigators selected studies independently, and a third investigator adjudicated disagreements. References of included studies were searched to find more relevant studies.

### 2.4 Data extraction and synthesis

Two investigators extracted data from each study, including authors, publication year, characters of subjects (disease status, age), sample size, study design, study location, study period, an average of markers level (TNF-α, IL-6, IL-8, fibrinogen), average levels of PM, exposure assessment methods, effect estimates (percent change, coefficient(β), relative change, fold change) and standard error or a 95% confidence interval. The data extraction was performed by two investigators and any disagreements were adjudicated by a third investigator.

We used the percent change as effect estimates. All estimates were converted into percent change of a 10 μg/m^3^ PM increase_._ Beta-coefficients from linear regression models were normalized using an equation β×10÷M×100%to calculate the percent change, and another equation[(β±1.96×SE)×10÷M]×100% to calculate 95% confidence intervals (CIs) (Yang et al., 2015), where β represents the regression coefficient, M represents the mean of markers level, and SE represents the standard error associated with β. Stata software (version 12.0; Stata Corp, U.S.) was used to conduct the meta-analysis.

### 2.5 Risk of bias evaluation

The OHAT tool, with some modification, was applied to evaluate risk of bias (Rooney et al., 2014). We considered some related reviews when formulating standards for the risk of bias used in this study (Supplemental Table S2) (Kirrane et al., 2019; Luben et al., 2017; Rooney et al., 2014). We assessed the following aspects: selection bias, disease misclassification, exposure assessment, confounding, detection bias, and selective reporting. Each aspect is rated as “high”, “probably high”, “probably low”, “low”, or “not applicable” based on specific criteria.

### 2.6 Statistical analysis

#### 2.6.1 Meta-analysis

Meta-analyses were conducted only when four or more eligible studies examined the association between the same pollutant and the same marker (Vrijheid et al., 2011). When studies reported the data of multi-pollutant models and single-pollutant models, we only analyzed the data of single-pollutant models. If only subgroup data were available in the study, then all subgroup results were included. When some studies provided several adjusted models, we used the “main model” or fully-adjusted model in our meta-analysis. If multiple lags were reported, we chose one based on the following criteria: (1) the lag that the investigators focused on or stated as a priority; (2) the lag that was statistically significant; (3) the lag with the largest effect estimate (Atkinson et al., 2012). In addition, for short-term studies, we pooled the effect estimates according to lag patterns when four or more estimates were available.

Meta-analyses based on the random-effects model were conducted to estimate the association between PM and inflammation and blood coagulation markers. I^2^, representing the proportion of heterogeneity in the total variation of effect, was used to quantify the heterogeneity among included studies. I^2^ values in the range of 50–100% indicate large or extreme heterogeneity (Higgins et al., 2003).

#### 2.6.2 Subgroup analysis

The heterogeneity among all included studies exists due to the differences in population characteristics, sample size, study designs, exposure assessment techniques, study locations, and pollution levels. To confirm the potential confounders, we performed subgroup analyses by disease status (general population or patients) (Liu et al., 2019), age (<60 years or ≥60 years) (Schneider et al., 2010), gender proportion (male≤50% or male >50%) (Clougherty, 2010), sample size (<1000 or ≥1000) (Liu et al., 2019), study design (panel study, cross-sectional study, others (time-series study, case-crossover study, semi-experimental design)), study location (Europe, Asia or North America), PM level (low or high according to WHO guidelines) (Krzyzanowski and Cohen, 2008), and exposure assessment techniques (fixed site monitors or others).

#### 2.6.3 Meta-regression, sensitivity analyses, and publication bias

To investigate the source of heterogeneity, we performed a meta-regression analysis (Higgins et al., 2011). Factors included disease status, age, gender proportion, sample size, study design, study location, average level of pollutants, and exposure assessment techniques.

Each study was removed in turn to investigate the sensitivity of pooled results. The assessment and correction of publication bias were based on Begg’s and Egger’s test (Egger et al., 1997) and “trim-and-fill” analysis.

## 3 Results

### 3.1 Study characteristics

Fig. 1 shows the selection process of literature. We identified 44 studies from citations screened (Chen et al., 2018; Chuang et al., 2007; Cole et al., 2018; Croft et al., 2017; Dadvand et al., 2014; Delfino et al., 2010; Deng et al., 2020; Dubowsky et al., 2006; Emmerechts et al., 2012; Forbes et al., 2009; Green et al., 2016; Habre et al., 2018; Hajat et al., 2015; Hassanvand et al., 2017; Hildebrandt et al., 2009; Hoffmann et al., 2009; Huttunen et al., 2012; Kumarathasan et al., 2018; Lanki et al., 2015; Lee et al., 2018; Liao et al., 2005; Mirowsky et al., 2015; Pekkanen et al., 2000; Pope et al., 2016; Puett et al., 2019; Rich et al., 2012; Ruckerl et al., 2007; Rückerl et al., 2014; Rudez et al., 2009; Schneider et al., 2010; Schwartz, 2001; Seaton et al., 1999; Steinvil et al., 2008; Strak et al., 2013; Su et al., 2017; Sullivan et al., 2007; Tsai et al., 2012; Viehmann et al., 2015; Wang et al., 2018; Wu et al., 2012; Zeka et al., 2006; Zhang et al., 2016, 2020; Zuurbier et al., 2011). Supplemental Table S3 provides the characteristics of included studies. Thirteen studies were conducted on patients with specific diseases, thirty on general populations, and one on patients and the general population. Sample size ranged from 22 to 20,000 for short-term studies, and from 242 to 25,000 for long-term studies. Seven studies assessed exposure using air pollution exposure models (land-use regression modeling, kriging interpolation modeling, and air dispersion modeling), and the rest based on fixed site or personal exposure measurement. Eighteen studies were performed in North America, sixteen in Europe, and ten in Asia. No study was conducted in South America or Africa.

**Fig. 1:**
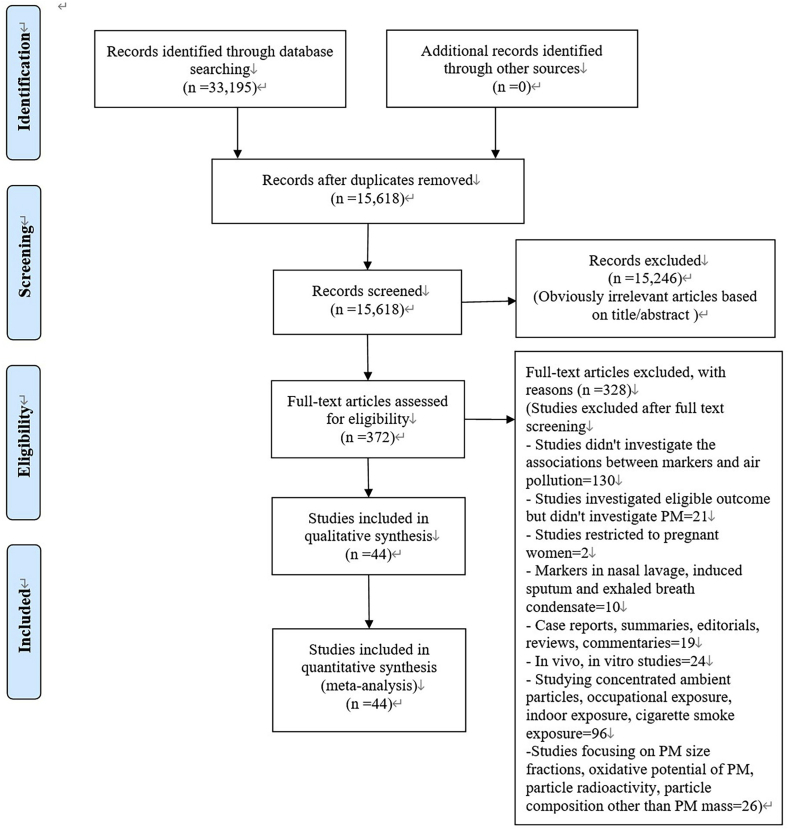
PRISMA 2009 flow diagram of study selection.

### 3.2 Risk of bias evaluation

**Fig. 2:**
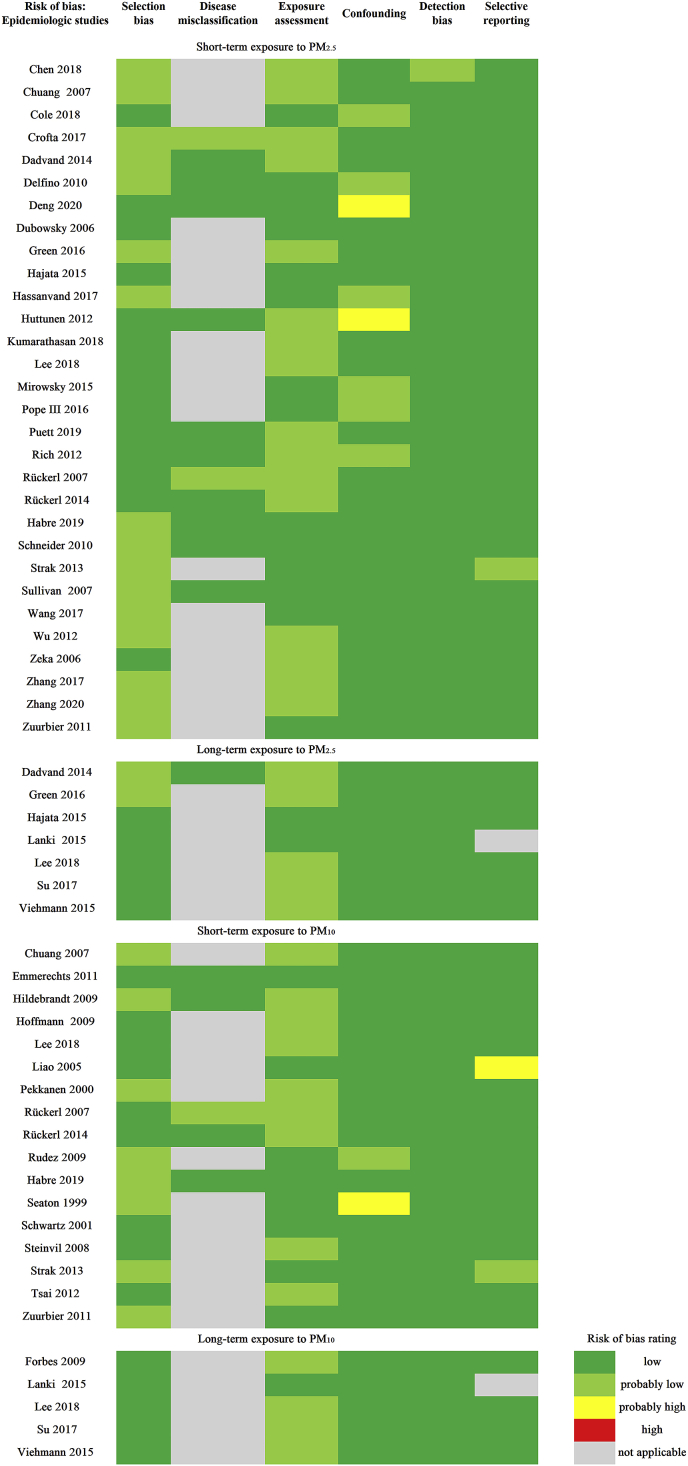
Risk of bias rating for each study.

The evaluation for risk of bias was shown in Fig. 2. Most of the studies were evaluated as ‘low’ or ‘probably low’ risk except four studies (Deng et al., 2020; Huttunen et al., 2012; Liao et al., 2005; Seaton et al., 1999). We considered that the included studies are of sufficient quality to evaluate the association between these markers and particulate air pollution. More details can be found in the supplementary materials (Table S4).

### 3.3 Associations between PM_2.5_ and markers

#### 3.3.1 Overall meta-analysis for PM_2.5_ and markers

**Fig. 3:**
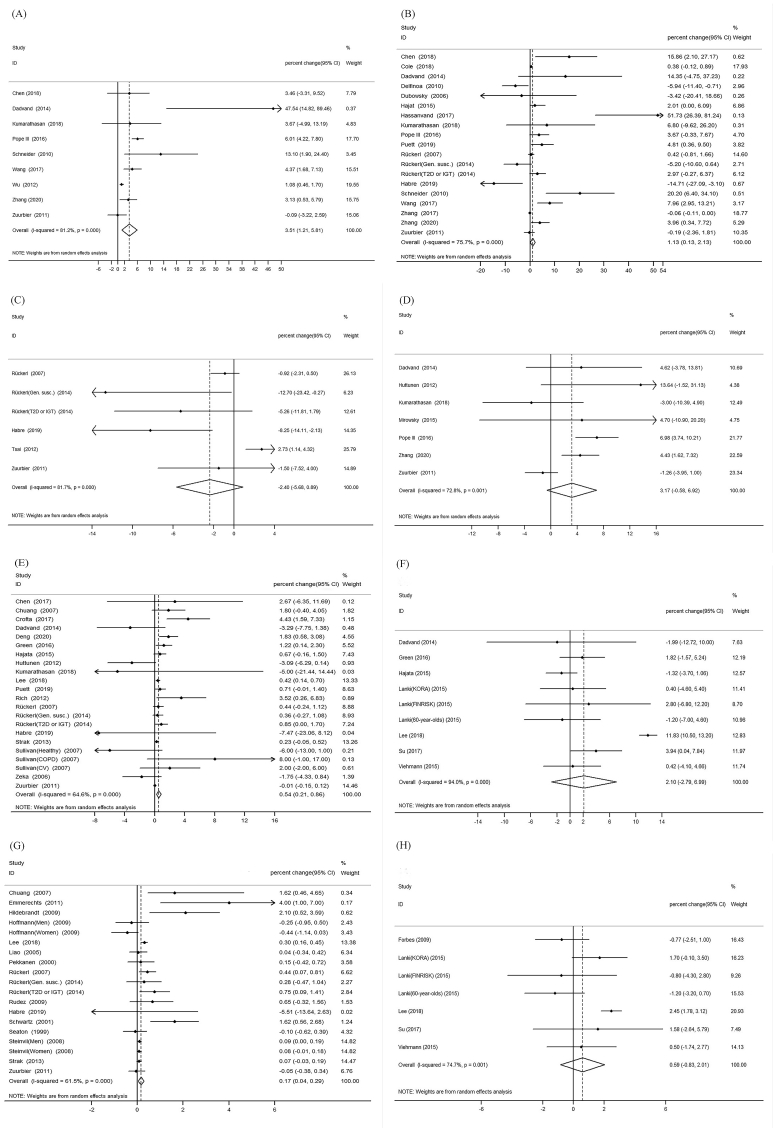
**Forest plot of the meta-analysis:** (A) short-term expose to PM_2.5_ and TNF-α (B) short-term exposure to PM_2.5_ and IL-6 (C) short-term exposure to PM_10_ an d IL-6 (D) short-term exposure to PM_2.5_ and IL-8 (E) short-term exposure to PM_2.5_ and fibrinogen (F) long-term exposure to PM_2.5_ and fibrinogen (G) short-term exposure to PM_10_ and fibrinogen (H) long-term exposure to PM_10_ and fibrinogen.

**Fig. 4:**
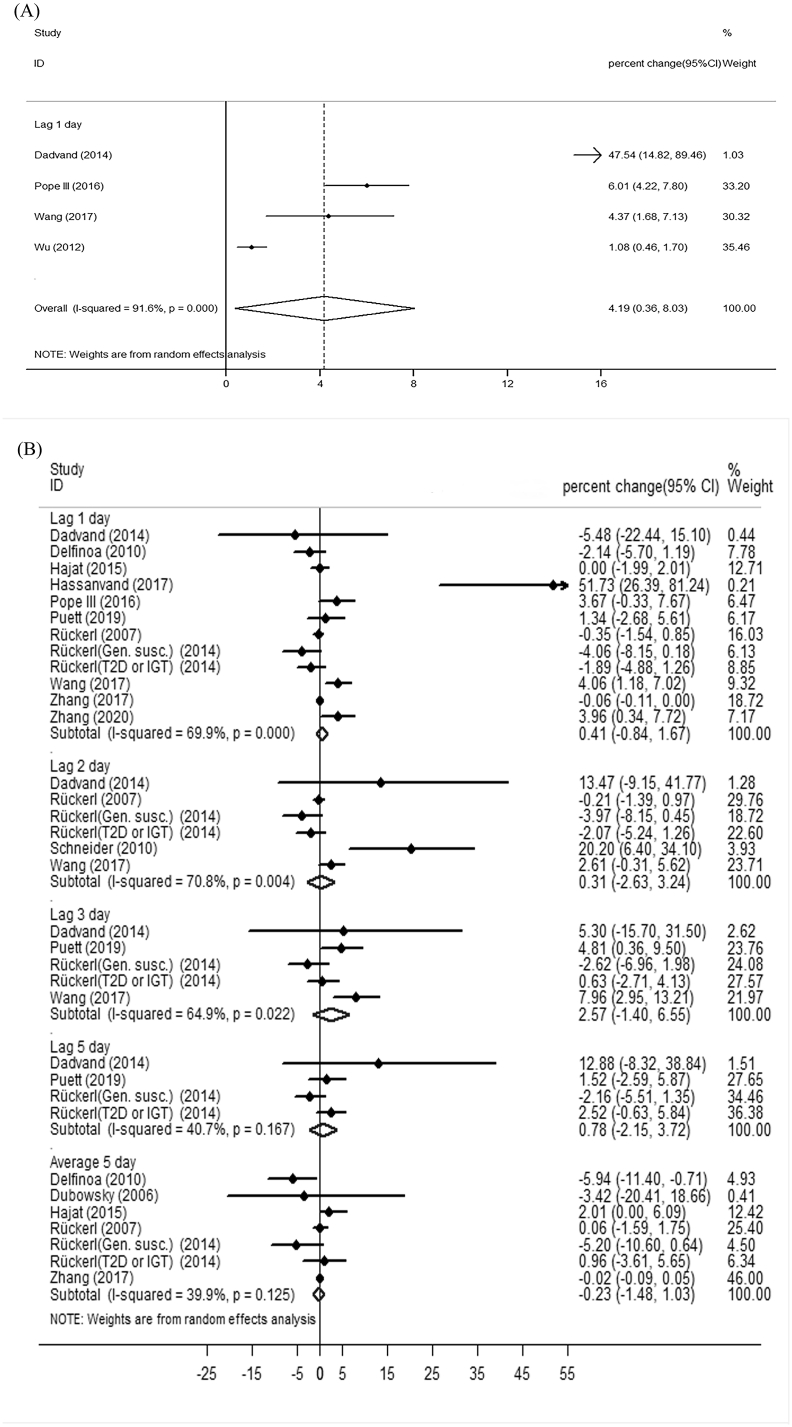

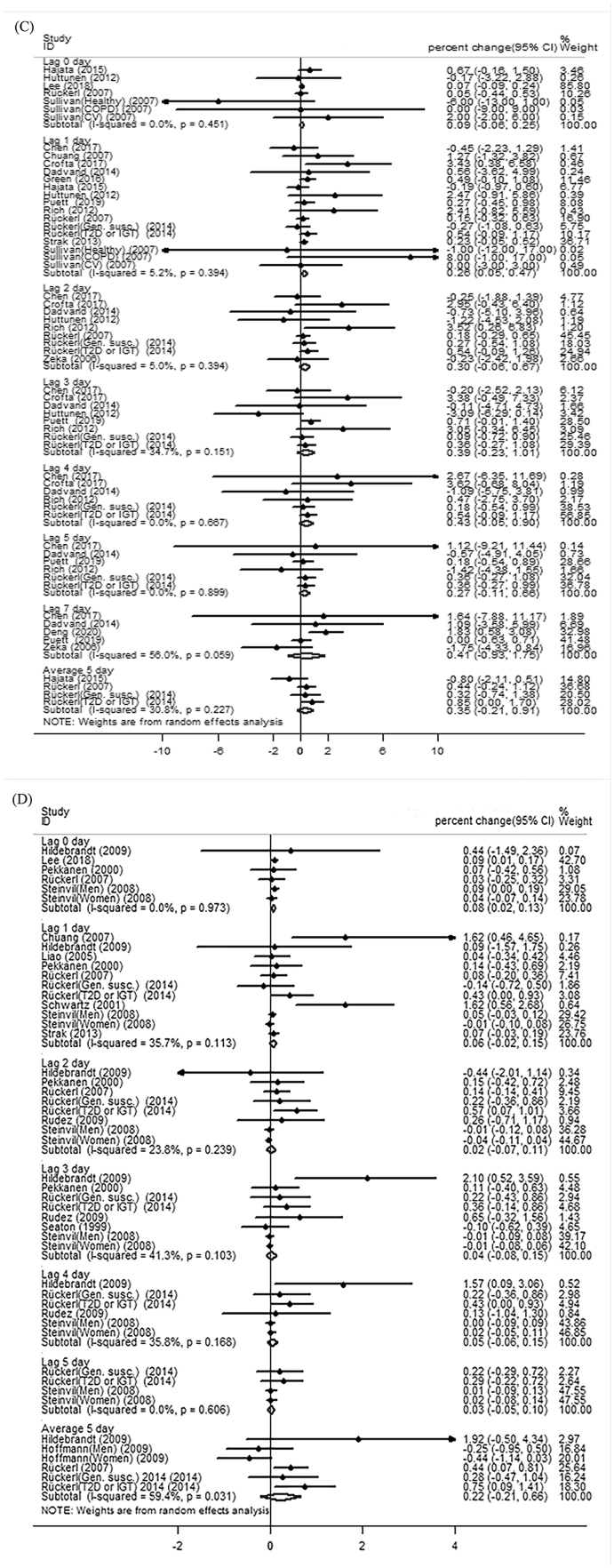
**Meta-analyses stratified by varying lag patterns** (A) short-term expose to PM_2.5_ and TNF-α (B) short-term exposure to PM_2.5_ and IL-6 (C) short-term exposure to PM_2.5_ and fibrinogen(D)short-term exposure to PM_10_ and fibrinogen.

Our meta-analysis showed significant changes of TNF-α and fibrinogen and insignificant changes of IL-6 and IL-8 with short-term PM_2.5_ exposure (Fig. 3(A), 3(E), 3(B), and 3(D)). For short-term PM exposure, the percent change of a 10 μg/m^3^ PM_2.5_ increase on TNF-α and fibrinogen were 3.51% (95% CI: 1.21%, 5.81%) and 0.54% (95% CI: 0.21%, 0.86%). Fibrinogen was not significantly associated with long-term PM_2.5_ exposure (Fig. 3(F)). Meta-analysis according to lag pattern showed that the percent change of a 10 μg/m^3^ PM_2.5_ increase on TNF-α (n = 4 studies) and fibrinogen (n = 12 studies) were 4.19% (95%CI: 0.36%, 8.03%) and 0.26% (95%CI: 0.05%, 0.47%) at lag 1 day respectively (Fig. 4).

#### 3.3.2 Subgroup-analysis for PM_2.5_ and markers

Sub-stratified analysis by study location showed that significant associations of PM_2.5_ with fibrinogen, TNF-α, and IL-6 in studies conducted in Asia compared to that conducted in Europe (Table 1). For example, we found a statistically significant association between short-term PM_2.5_ exposure and IL-6 in studies conducted in Asia (percent change = 11.65%, 95%CI: 3.02%, 20.28%), while an insignificant association in studies conducted in Europe (percent change = 0.32%, 95%CI: −1.61%, 2.25%) (Table 1).

**Table 1:** Subgroup analysis of percent change in inflammation and blood coagulation markers in association with a 10 μg/m^3^ increase in ambient PM concentration.

Biomarker	Subgroup	Exposure	Grouping criteria	Pooled percent-changes (95% CI)	P value	No. of effect estimates	No. of studies	Heterogeneity	
								*P*-value for heterogeneity	I^2^
Disease status									
Fibrinogen	PM_2.5_	Short-term	General population	0.31 (-0.01, 0.63)	0.061	9	9	0.005	64.00%
			Patients	0.88 (0.20, 1.55)	0.011	13	11	0.009	54.7%
		Long-term	General population	2.44 (-2.67, 7.54)	0.349	8	6	<0.001	94.60%
	PM_10_	Short-term	General population	0.11 (0.00, 0.21)	0.041	13	11	0.014	52.10%
			Patients	0.79 (0.15, 1.42)	0.015	6	5	0.031	59.40%
TNF-α	PM_2.5_	Short-term	General population	2.98 (0.85, 5.12)	0.006	7	7	<0.001	81.7%
IL-6	PM_2.5_	Short-term	General population	1.22(0.15, 2.28)	0.025	11	11	<0.001	76.4%
			Patients	0.29 (-3.44, 4.01)	0.881	8	7	<0.001	77.2%
Age									
Fibrinogen	PM_2.5_	Short-term	<60	0.32 (0.00, 0.64)	0.047	8	8	.041	52.2%
			≥60	0.57 (-0.40, 1.54)	0.253	12	10	0.003	60.90%
		Long-term	<60	2.28 (0.06,4.50)	0.044	4	3	0.724	0.00%
			≥60	−0.99 (-2.92, 0.95)	0.319	4	4	0.918	0.00%
	PM_10_	Short-term	<60	0.12 (-0.03, 0.26)	0.113	13	12	0.002	60.70%
			≥60	0.23 (-0.18, 0.65)	0.274	4	4	0.072	57.10%
TNF-α	PM_2.5_	Short-term	<60	2.98 (0.85, 5.12)	0.006	7	7	<0.001	81.7%
IL-6	PM_2.5_	Short-term	<60	2.47 (-0.17, 5.12)	0.067	11	11	<0.001	79.0%
			≥60	0.70 (-0.88, 2.29)	0.384	8	8	0.005	65.50%
Sex									
Fibrinogen	PM_2.5_	Short-term	male≤50%	0.41 (0.11,0.72)	0.008	7	7	0.211	28.50%
			male>50%	0.54 (-0.01, 1.10)	0.056	14	12	<0.001	65.0%
		Long-term	male≤50%	2.20 (-3.60, 8.00)	0.457	7	5	<0.001	95.30%
	PM_10_	Short-term	male≤50%	0.16 (-0.02,0.34)	0.084	9	9	<0.001	72.10%
			male>50%	0.25 (0.00,0.49)	0.046	9	8	0.026	53.90%
		Long-term	male≤50%	0.50 (-1.04, 2.03)	0.525	6	4	<0.001	79.00%
TNF-α	PM_2.5_	Short-term	male>50%	3.63 (0.27, 6.99)	0.034	6	6	<0.001	86.60%
IL-6	PM_2.5_	Short-term	male≤50%	1.86 (-1.31, 5.03)	0.250	7	7	0.001	72.8%
			male>50%	1.42 (-0.42, 3.27)	0.130	12	11	<0.001	76.5%
Sample size									
Fibrinogen	PM_2.5_	Short-term	<1000	0.36 (-0.12, 0.84)	0.14	16	13	0.001	60.70%
			≥1000	0.64 (0.33, 0.96)	<0.001	6	6	0.245	25.2%
		Long-term	≥1000	2.20 (-3.60, 8.00)	0.457	7	5	<0.001	95.30%
	PM_10_	Short-term	<1000	0.34 (0.00, 0.68)	0.05	10	9	0.005	62.00%
			≥1000	0.15 (0.01, 0.29)	0.031	9	7	0.004	65.00%
		Long-term	≥1000	0.50 (-1.04, 2.03)	0.525	6	4	<0.001	79.00%
TNF-α	PM_2.5_	Short-term	<1000	3.51 (1.21, 5.81)	0.003	9	9	<0.001	81.2%
IL-6	PM_2.5_	Short-term	<1000	1.04 (-0.15, 2.24)	0.088	16	15	<0.001	77.8%
Study design									
Fibrinogen	PM_2.5_	Short-term	Panel study	0.62 (0.22, 1.02)	0.002	13	10	0.087	37.10%
			Cross-sectional study	0.33 (-2.13, 2.80)	0.790	4	4	0.005	76.6%
			Others	0.25 (-0.17, 0.68)	0.241	5	5	0.022	65.2%
		Long-term	Panel study	3.27 (-4.55, 11.09)	0.413	4	4	<0.001	97.30%
			Cross-sectional study	1.65 (-0.89, 4.20)	0.203	5	3	0.568	0.00%
	PM_10_	Short-term	Panel study	0.48 (0.17, 0.78)	0.003	9	8	0.024	54.70%
			Cross-sectional study	0.07 (-0.07,0.21)	0.323	7	5	0.06	50.30%
		Long-term	Cross-sectional study	−0.01 (-1.33, 1.31)	0.99	5	3	0.175	37.00%
TNF-α	PM_2.5_	Short-term	Panel study	4.06 (1.24, 6.89)	0.005	5	5	<0.001	88.5%
IL-6	PM_2.5_	Short-term	Panel study	1.72 (-0.11, 3.54)	0.065	12	11	<0.001	79.1%
			Cross-sectional study	5.24 (0.77, 9.71)	0.021	2	2	0.384	0.0%
			Others	0.28 (-2.72, 3.29)	0.853	5	5	0.013	68.50%
Study location									
Fibrinogen	PM_2.5_	Short-term	Europe	0.21 (-0.10, 0.51)	0.189	7	6	0.033	56.10%
			Asia	1.09 (0.06, 2.13)	0.038	4	4	0.104	51.4%
			North America	1.04 (0.08, 2.00)	0.034	11	9	0.021	52.5%
		Long-term	Europe	0.13 (-2.54, 2.79)	0.926	5	3	0.955	0.00%
	PM_10_	Short-term	Europe	0.19 (-0.05, 0.42)	0.12	12	10	0.004	59.90%
			Asia	0.15 (0.03, 0.28)	0.019	4	3	0.024	68.20%
		Long-term	Europe	−0.02 (-1.18, 1.14)	0.968	5	3	0.199	33.30%
TNF-α	PM_2.5_	Short-term	Asia	2.56 (0.62, 4.49)	0.01	4	4	0.055	60.6%
IL-6	PM_2.5_	Short-term	Europe	0.32 (-1.61, 2.25)	0.745	5	4	0.087	50.70%
			Asia	11.65 (3.02, 20.28)	0.008	4	4	0.002	79.6%
			North America	0.54 (-0.50, 1.57)	0.310	10	10	<0.001	71.4%
Pollution level									
Fibrinogen	PM_2.5_	Short-term	Low	0.62 (0.19, 1.05)	0.004	16	13	0.015	48.8%
			High	0.83 (-0.44, 2.09)	0.201	5	5	0.035	61.2%
		Long-term	High	2.47 (-3.08, 8.02)	0.384	7	7	<0.001	95.20%
	PM_10_	Short-term	Low	0.28 (0.04, 0.52)	0.021	16	14	0.001	61.60%
		Long-term	Low	−0.61 (-1.69, 0.46)	0.264	4	3	0.723	0.00%
TNF-α	PM_2.5_	Short-term	High	2.94 (0.43, 5.44)	0.022	5	5	0.009	70.4%
IL-6	PM_2.5_	Short-term	Low	0.38 (-0.50, 1.26)	0.401	12	11	<0.001	68.7%
			High	11.71 (3.82, 19.60)	0.004	5	5	0.004	73.8%
Exposure assessment									
Fibrinogen	PM_2.5_	Short-term	Fixed site	0.65 (0.27, 1.03)	0.001	19	16	0.004	52.2%
			Others	0.18 (-0.58, 0.95)	0.639	3	3	0.05	66.5%
		Long-term	Others	1.34 (-0.86, 3.54)	0.232	6	4	0.674	0.00%
	PM_10_	Short-term	Fixed site	0.20 (0.08, 0.32)	0.001	15	13	0.002	59.90%
			Others	−0.08 (-0.68, 0.53)	0.803	4	3	0.034	65.40%
		Long-term	Others	0.07 (-0.99, 1.14)	0.891	6	4	0.259	23.30%
TNF- α	PM_2.5_	Short-term	Fixed site	3.91 (1.01, 6.80)	0.008	6	6	<0.001	84.2%
IL-6	PM_2.5_	Short-term	Fixed site	1.28 (-0.61, 3.18)	0.184	13	12	<0.001	78.4%
			Others	2.09 (-0.30, 4.48)	0.087	6	6	0.016	64.3%

Abbreviations: PM_2.5_:particulate matter with aerodynamic diameter equal to or less than 2.5 μm; PM_10_: particulate matter with aerodynamic diameter equal to or less than 10 μm; TNF- α: tumor necrosis factor-alpha, IL-6: interleukin-6; NA: not applicable.

#### 3.3.3 Studies not included in meta-analysis

There are only one, two, and one studies investigated the associations of long-term PM_2.5_ exposure with TNF-α (Dadvand et al., 2014), IL-6 (Dadvand et al., 2014; Hajat et al., 2015), and IL-8 (Dadvand et al., 2014), which was too small to permit us to perform a meta-analysis.

### 3.4 Associations between PM_10_ and markers

#### 3.4.1 Overall meta-analysis for PM_10_ and markers

Our meta-analysis showed a significant short-term association between PM_10_ and fibrinogen (Fig. 3(G); n = 16 studies) and an insignificant long-term association between PM_10_ and fibrinogen (Fig. 3(H); n = 5 studies). The percent change of a 10 μg/m^3^ PM_10_ increase on fibrinogen was 0.17% (95% CI: 0.44%, 0.29%). The pooled estimate of IL-6 with short-term PM_10_ exposure was not significant (Fig. 3(C); n = 5 studies). Meta-analysis stratified by lag pattern showed a 0.08% (95%CI: 0.02%, 0.13%) increase in fibrinogen (n = 5 studies) per 10 μg/m^3^ exposure to PM_10_ at lag 0 day (Fig. 4).

#### 3.4.2 Subgroup analysis for PM_10_ and markers

Sub-stratified analysis by exposure assessment technique, subjects, study location, and study design showed that a significant or stronger short-term association between PM_10_ and fibrinogen in studies assigning exposure based on fixed-site, for patients, conducted in Asia and panel design compared to that assigning exposure using other methods, for the general population, performed in Europe and cross-sectional design (Table 1). For example, we found a significant short-term association between PM_10_ and fibrinogen in studies for patient (percent change = 0.79%, 95%CI: 0.15%, 1.42%), followed by general population (percent change = 0.11%, 95%CI: 0.00%, 0.21%).

#### 3.4.3 Studies not included in meta-analysis

There are only two studies investigated the associations of short-term PM_10_ exposure with TNF-α (Tsai et al., 2012; Zuurbier et al., 2011) and IL-8 (Mirowsky et al., 2015; Zuurbier et al., 2011), which was too small to permit us to perform a meta-analysis.

### 3.5 Meta-regression analysis, sensitivity analyses and publication bias

**Fig. 5:**
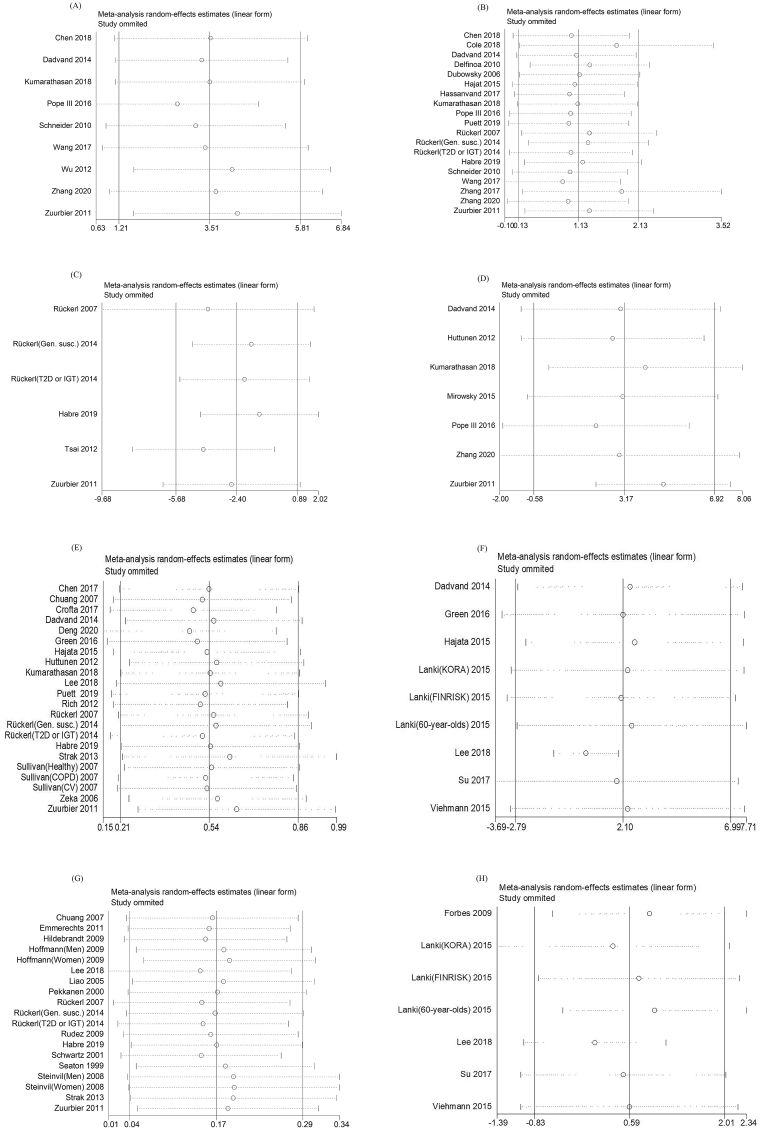
Sensitivity analyses for the association between PM and inflammation and blood coagulation level change excluding studies one by one (A) short-term expose to PM_2.5_ and TNF-α (B) short-term exposure to PM_2.5_ and IL-6 (C) short-term exposure to PM_10_ an d IL-6 (D) short-term exposure to PM_2.5_ and IL-8 (E) short-term exposure to PM_2.5_ and fibrinogen (F) long-term exposure to PM_2.5_ and fibrinogen (G) short-term exposure to PM_10_ and fibrinogen (H) long-term exposure to PM_10_ and fibrinogen.

**Fig. 6:**
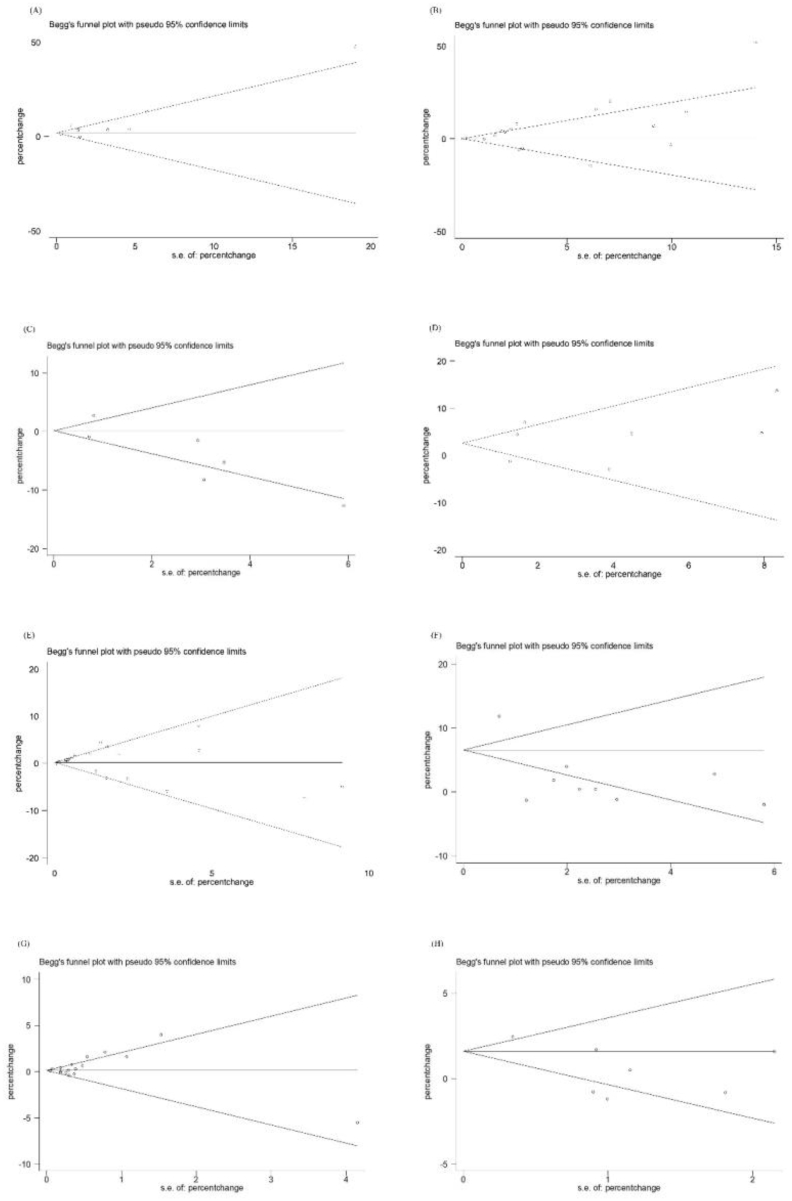
Begg’s funnel plots of publication bias analyses (A) short-term expose to PM2.5 and TNF-α (B) short-term exposure to PM2.5 and IL-6 (C) short-term exposure to PM10 an d IL-6 (D) short-term exposure to PM2.5 and IL-8 (E) short-term exposure to PM2.5 and fibrinogen (F) long-term exposure to PM2.5 and fibrinogen (G) short-term exposure to PM10 and fibrinogen (H) long-term exposure to PM10 and fibrinogen (s.e.: standard error).

Meta-regression analysis showed air pollutants levels, age, study location, disease status, and study design may be the source of heterogeneity (Table 2). Sensitivity analyses supported the results of meta-analyses for all inflammation and blood coagulation markers (Fig. 5). Begg’s funnel plots of PM_2.5_ and TNF-α, IL-8 show general symmetry (Fig. 6). Also, *P*-values of Begg’s and Egger’s tests indicated no publication bias of analyses on PM_2.5_ and TNF-α, IL-8 (Table 3). For IL-6, the *P*-value of Egger’s test in studies reporting short-term association between PM_2.5_ and IL-6 was 0.02. Trim-and-fill analysis shows the change in the overall analysis for studies reporting the short-term association between PM_2.5_ and IL-6 is 0.90% (95%CI: −0.02%, 2.00%) (Table 3, Figure S1 (A)). For short-term PM exposure, we did not observe publication bias of analyses on fibrinogen and PM_2.5_, PM_10_. However, the *P*-value of Egger’s test in studies reporting long-term association for PM_2.5_-fibrinogen was 0.05 (n = 7 studies). Trim-and-fill analysis shows no change in the overall analysis for studies reporting the long-term association for PM_2.5_-fibrinogen (Figure S1(B)).

**Table 2:** Meta-regression analysis by potential modifier.

Biomarker	Subgroup	Exposure	Grouping criteria	No. of effect estimates	No. of studies	Meta-regression		
						Coef.	P value	I^2^
Disease status								
Fibrinogen	PM_2.5_	Short-term	General population	9	9	Reference	0.196	58.96%
			Patients	13	11	0.45 (-0.25, 1.16)		
		Long-term	General population	8	6	Reference	0.563	94.63%
			Patients	1	1	−4.56 (-22.35,13.22)		
	PM_10_	Short-term	General population	13	11	Reference	0.051	54.53%
			Patients	6	5	0.46 (0.00,0.93)		
TNF-α	PM_2.5_	Short-term	General population	7	7	Reference	0.064	80.45%
			Patients	2	2	13.30 (-1.04, 27.64)		
IL-6	PM_2.5_	Short-term	General population	11	11	Reference	0.377	76.73%
			Patients	8	7	−3.20 (-10.65, 4.25)		
Age								
Fibrinogen	PM_2.5_	Short-term	<60	8	8	−0.20 (-1.23, 0.82)	0.8007	59.89%
			≥60	12	10	Reference		
			NA	2	2	0.15 (-1.17, 1.48)		
		Long-term	<60	4	3	3.27 (-0.41,6.94)	0.0001	0.00%
			≥60	4	4	Reference		
			NA	1	1	12.81 (9.86, 15.76)		
	PM_10_	Short-term	<60	13	12	−0.18 (-0.61, 0.25)	0.1795	57.60%
			≥60	4	4	Reference		
			NA	2	2	0.03 (-0.45, 0.51)		
		Long-term	<60	3	2	1.38 (-3.49, 6.25)	0.6703	71.48%
			≥60	2	2	Reference		
			NA	2	2	1.45 (-3.23, 6.14)		
TNF-α	PM_2.5_	Short-term	<60	7	7	−13.30 (-27.64, 1.04)	0.064	80.45%
			≥60	2	2	Reference		
IL-6	PM_2.5_	Short-term	<60	11	11	1.22 (-6.53, 8.97)	0.744	75.00%
			≥60	8	8	Reference		
Sex								
Fibrinogen	PM_2.5_	Short-term	male≤50%	7	7	Reference	0.3688	58.26%
			male>50%	14	12	−0.03 (-0.80, 0.73)		
			NA	1	1	1.38 (-0.69, 3.46)		
		Long-term	male≤50%	7	5	Reference	0.953	94.59%
			male>50%	2	2	−0.29 (-11.22,10.65)		
	PM_10_	Short-term	male≤50%	9	9	Reference	0.7892	65.24%
			Male>50%	9	8	0.06 (-0.34,0.46)		
			NA	1	1	−0.26 (-1.22,0.71)		
		Long-term	male≤50%	6	4	Reference	0.712	78.96%
			male>50%	1	1	1.04 (-5.79,7.86)		
TNF-α	PM_2.5_	Short-term	male≤50%	3	3	Reference	0.88	81.51%
			male>50%	6	6	−0.45 (-7.31, 6.41)		
IL-6	PM_2.5_	Short-term	male≤50%	7	7	Reference	0.701	75.30%
			male>50%	12	11	1.57 (-6.90, 10.03)		
Sample size								
Fibrinogen	PM_2.5_	Short-term	<1000	16	13	−0.41 (-1.07, 0.24)	0.204	55.37%
			≥1000	6	6	Reference		
		Long-term	<1000	2	2	−0.29 (-11.22,10.65)	0.953	94.59%
			≥1000	7	5	Reference		
	PM_10_	Short-term	<1000	10	9	0.04 (-0.36,0.43)	0.844	63.45%
			≥1000	9	7	Reference		
		Long-term	<1000	1	1	1.04 (-5.79,7.86)	0.712	78.96%
			≥1000	6	4	Reference		
IL-6	PM_2.5_	Short-term	<1000	16	15	0.10 (-9.46, 9.65)	0.983	76.17%
			≥1000	3	3	Reference		
Study design								
Fibrinogen	PM_2.5_	Short-term	Panel study	13	10	Reference	0.4046	56.21%
			Cross-sectional study	4	4	0.09 (-1.11, 1.30)		
			Others	5	5	−0.39 (-1.03, 0.25)		
		Long-term	Panel study	4	4	Reference	0.521	93.95%
			Cross-sectional study	5	3	−2.36 (-10.63,5.91)		
	PM_10_	Short-term	Panel study	9	8	Reference	0.0476	49.95%
			Cross-sectional study	7	5	−0.26 (-0.47, −0.05)		
			Others	3	3	−0.27 (-0.52, −0.03)		
		Long-term	Panel study	2	2	Reference	0.167	44.31%
			Cross-sectional study	5	3	−1.82 (-4.71,1.08)		
TNF-α	PM_2.5_	Short-term	Panel study	5	5	Reference	0.1101	83.36%
			Cross-sectional study	1	1	43.68 (-3.27, 90.63)		
			Others	3	3	−2.47 (-8.03, 3.09)		
IL-6	PM_2.5_	Short-term	Panel study	12	11	Reference	0.7533	75.80%
			Cross-sectional study	2	2	4.02 (-11.07, 19.11)		
			Others	5	5	−1.85 (-11.48, 7.78)		
Study location								
Fibrinogen	PM_2.5_	Short-term	Europe	7	6	−0.50 (-1.36, 0.36)	0.2133	53.33%
			Asia	4	4	Reference		
			North America	11	9	0.18 (-0.88, 1.24)		
		Long-term	Europe	5	3	−8.50 (-15.51,-1.49)	0.0447	64.46%
			Asia	2	2	Reference		
			North America	2	2	−8.49 (-15.94,-1.04)		
	PM_10_	Short-term	Europe	12	10	−0.01 (-0.47,0.45)	0.9245	65.46%
			Asia	4	3	Reference		
			North America	3	3	0.14 (-0.68,0.96)		
		Long-term	Europe	5	3	−2.37 (-4.84,0.09)	0.056	18.79%
			Asia	2	2	Reference		
TNF-α	PM_2.5_	Short-term	Europe	2	2	−2.08 (-9.06, 4.90)	0.2211	61.54%
			Asia	4	4	Reference		
			North America	3	3	3.73 (-1.87, 9.32)		
IL-6	PM_2.5_	Short-term	Europe	5	4	−8.35 (-17.78, 1.07)	0.1641	70.57%
			Asia	4	4	Reference		
			North America	10	10	−7.68 (-16.48, 1.12)		
Pollution level								
Fibrinogen	PM_2.5_	Short-term	Low	16	13	0.11 (-0.68, 0.91)	0.2968	52.05%
			High	5	5	Reference		
			NA	1	1	−0.47 (-1.40, 0.46)		
		Long-term	Low	2	1	−2.32 (-13.19,8.55)	0.629	94.41%
			High	7	7	Reference		
	PM_10_	Short-term	Low	16	14	0.17 (-0.03,0.36)	0.086	56.55%
			High	3	2	Reference		
		Long-term	Low	4	3	−2.95 (-4.58, −1.33)	0.006	0.00%
			High	3	3	Reference		
TNF-α	PM_2.5_	Short-term	Low	2	2	4.53 (-8.98, 18.03)	0.7233	78.10%
			High	5	5	Reference		
			NA	2	2	0.18 (-7.46, 7.83)		
IL-6	PM_2.5_	Short-term	Low	12	11	−8.63 (-16.84, −0.41)	0.1147	69.94%
			High	5	5	Reference		
			NA	2	2	−7.40 (-17.98, 3.18)		
Exposure assessment								
Fibrinogen	PM_2.5_	Short-term	Fixed site	19	16	Reference	0.300	54.21%
			Other	3	3	−0.42 (-1.25, 0.41)		
		Long-term	Fixed site	3	3	Reference	0.362	93.36%
			Other	6	4	−3.36 (-11.51,4.79)		
	PM_10_	Short-term	Fixed site	15	13	Reference	0.195	60.97%
			Other	4	3	−0.32 (-0.83,0.18)		
		Long-term	Fixed site	1	1	Reference	0.071	23.26%
			Other	6	4	−2.37 (-5.03, 0.29)		
TNF-α	PM_2.5_	Short-term	Fixed site	6	6	Reference	0.713	83.36%
			Other	3	3	−1.14 (-8.18, 5.90)		
IL-6	PM_2.5_	Short-term	Fixed site	13	12	Reference	0.774	75.59%
			Other	6	6	1.14 (-7.10, 9.39)		

Abbreviations: PM_2.5_:particulate matter with aerodynamic diameter equal to or less than 2.5 μm; PM_10_: particulate matter with aerodynamic diameter equal to or less than 10 μm; TNF-α: tumor necrosis factor-alpha, IL-6: interleukin-6; NA: not applicable.

**Table 3:** Publication bias analyses.

Biomarker	Pollutant	Exposure	Begg’s test (*P*-value)	Eggr’s test (*P*-value)	Trim-and-fill estimate Pooled %-changes (95% CI)
TNF-α	PM_2.5_	Short-term	0.47	0.07	–
IL-6	PM_2.5_	Short-term	0.18	0.02	0.90 (-0.20, 2.00)
	PM_10_	Short-term	0.45	0.15	–
IL-8	PM_2.5_	Short-term	0.76	0.64	–
fibrinogen	PM_2.5_	Short-term	0.69	0.09	–
		Long-term	0.47	0.05	–
	PM_10_	Short-term	0.17	0.11	–
		Long-term	0.76	0.07	–

Abbreviations: PM_2.5_:particulate matter with aerodynamic diameter equal to or less than 2.5 μm; PM_10_: particulate matter with aerodynamic diameter equal to or less than 10 μm; TNF-α: tumor necrosis factor-alpha, IL-6: interleukin-6.

## 4 Discussion

To our knowledge, we conducted this first review to comprehensively summarize and quantitatively analyze short- and long-term association of PM_2.5_/PM_10_ with key inflammation and blood coagulation markers. Our meta-analysis showed significant short-term associations between PM_2.5_ and fibrinogen (percent change = 0.44%, 95%CI: 0.11%, 0.77%) and PM_10_-fibrinogen (percent change = 0.17%, 95%CI: 0.04%, 0.29%). For short-term PM exposure, the overall analysis showed the percent change of a 10 μg/m^3^ PM_2.5_ increase on TNF-α was 3.67% (95%CI: 0.97%, 6.36%). However, in long-term studies, the pooled estimates of fibrinogen with PM_2.5_, and PM_10_ were insignificant.

Given the important role of TNF-α and fibrinogen for inflammation and coagulation cascade in cardiovascular disease, our results support that short-term PM exposure might cause adverse effects on the human body through inflammation and coagulation cascade. When human bodies are exposed to particulate air pollution, particles can cause an acute-phase response and inflammation indicated by increments of fibrinogen and inflammatory cytokines (Fiordelisi et al., 2017; Franchini and Mannucci, 2007). Particles can cause an acute-phase response when it reach the bronchi and alveolar cells (Brook et al., 2010). Fibrinogen, a marker of acute-phase response, is not only a blood coagulation but also play a role in inflammation (Kattula et al., 2017). Fibrinogen can activate inflammation, leading to the release of inflammatory cytokines, such as TNF-α (Jensen et al., 2007). TNF-α is an inflammatory marker and involved in the development of atherosclerosis (Popa et al., 2007). Inflammatory cytokines due to air pollution exposure can also trigger fibrinogen production (Mutlu et al., 2007). Fibrinogen due to air pollution may increases plasma viscosity and induces platelet adhesion and aggregation, which could enhance coagulation potential and increase the risk of venous thrombosis leading to the development of cardiovascular disease (Brook et al., 2010; Tousoulis et al., 2011).

Not all studies included in this review showed significant changes of TNF-α and fibrinogen with short-term PM exposure. Zuurbier et al. reported insignificant changes of TNF-α and fibrinogen with PM_2.5_ exposure during commuting, which may be due to exercise (Zuurbier et al., 2011). Exercise is considered to be a method in controlling the expression of inflammation markers (Neves Miranda et al., 2015) and coagulation markers (Kupchak et al., 2017). Exercise has an effect on anti-inflammatory, including reduced IL-6 and TNF-α (Woods et al., 2009). Exercise also has effects on coagulation and fibrinolysis, which could significant degrade fibrinogen (El-Sayed et al., 2004). Moreover, the study by Zuurbier only measured blood markers at lag 6 h, which did not allow more time windows of response (Zuurbier et al., 2011). If we observe multiple time windows, we could find more changes of markers.

The short-term associations of exposure to PM with inflammation and blood coagulation markers are different at lag length, and such effects might be different in populations. A study conducted in COPD patients reported that the percent change of a 10.8 μg/m^3^ PM_2.5_ increase on TNF-α was 52.2% (95%CI: 16.1%, 99.4%) at lag 1 day (Dadvand et al., 2014). Among healthy college students, Wang et al. reported that, at lag 1 day, the percent change of a 10 μg/m^3^ PM_2.5_ increase on TNF-α was 4.37% (95% CI: 11.68%, 7.13%) (Wang et al., 2018). Also, some studies reported the significant change of these markers at a longer lag interval. Among patients undergoing cardiac rehabilitation, Rich et al. found that the percent change of a 6.5 μg/m^3^ PM_2.5_ increase on fibrinogen was 0.082 g/L (95%CI: 0.006 g/L, 0.159 g/L) at lag 2 day (Rich et al., 2012). Study on male patients showed that the percent change of a 11.43 μg/m^3^ PM_10_ increase on fibrinogen were 2.4% (95%CI: 0.6%, 4.1%) and 1.8% (95%CI: 0.1%, 3.5%) at lag 3 day and lag 4 day, respectively (Hildebrandt et al., 2009). Rückerl et al. reported a significant change of fibrinogen with 5-day average PM_2.5_ exposure in impaired glucose tolerance patients or type 2 diabetes mellitus patients, but not in genetically susceptible subjects (Rückerl et al., 2014). To investigate the lag effect of PM exposure on changes of these markers, we conducted meta-analyses according to lag patterns. Meta-analysis stratified by lag pattern showed that the percent change of a 10 μg/m^3^ PM_2.5_ increase on TNF-α and fibrinogen were 4.19% (95% CI: 0.36%, 8.03%) and 0.26% (95% CI: 0.02%, 0.51%) at lag 1 day respectively, and 0.08% (95%CI: 0.02%, 0.13%) higher fibrinogen levels per 10 μg/m^3^ exposure to PM_10_ at lag 0 day.

Subgroup analysis by PM concentrations showed that significant associations of short-term PM_2.5_ exposure with TNF-α and IL-6 in higher PM levels. Interestingly, it was found significant associations of short-term PM_2.5_ and PM_10_ exposure with fibrinogen in lower PM levels. Liang et al. also reported that the change of von Willebrand factor was more sensitive in the subgroup with PM_2.5_ < 25 μg/m^3^ (Liang et al., 2020). Similarly, the association between short-term PM_2.5_ exposure and C-reactive protein was greater in the subgroup with PM_2.5_ lower than 25 μg/m^3^ (Liu et al., 2019).

Subgroup analysis by study location showed that the change of inflammation and blood coagulation markers were significant in Asia. For example, we found a significant short-term association between PM_2.5_ and IL-6 in studies conducted in Asia (percent change: 19.82%, 95%CI: 2.94%, 36.70%), but an insignificant association in studies conducted in Europe (percent change: 0.32%, 95%CI: −1.61%, 2.25%) or North America (percent change: 0.32%, −0.68%, 1.32%). We also found significant pooled estimates of fibrinogen with PM_2.5_ and PM_10_ exposure in studies conducted in Asia, but not in Europe or North America. A study conducted in 10 cities around the world found that the cities located in Europe (except Milan) all met the EU PM_2.5_ annual mean standard (25 μg/m^3^), while the cites located in Asia have the highest PM_2.5_ annual mean concentrations (de Jesus et al., 2019). Pollution level in Asia is higher than in Europe, which may contribute to this finding. The lack of study in Africa is concerning because these areas may have a more significant impact (Li et al., 2018). In our meta-analysis, there is no study conducted in Africa.

There are differences in the sources of particulate matter in different regions, which may be the main reason for the differences in biomarkers. Some countries in Asia have serious industrial pollution (Zhang et al., 2019), while in Europe, the proportion of particulate matter caused by industrial emissions is relatively small. The main sources of particulate matter are vehicular source, crustal source, sea-salt source and secondary aerosol source (Viana et al., 2008). A study conducted in France showed that the highest source of PM_10_ is secondary inorganic aerosols (28%), while the lowest source is heavy oil combustion (4%) (Waked et al., 2014). A study conducted in China reported that the main sources of PM_2.5_ are coal combustion, industrial emissions and vehicular exhaust (Zhang et al., 2019). Moreover, the pollutant concentration in Asia has been above the WHO threshold for longer than in Europe, which may also contribute to the continental differences (de Jesus et al., 2019).

The variation of components in different regions may be a reason for inconsistent findings among studies (Steenhof et al., 2011). In China, Wu et al. found that an increase of 3.91% (95%CI: 0.31%, 7.63%) in fibrinogen per 0.51 μg/m^3^ exposure to the iron of PM_2.5_ at lag 1 day among healthy adults (Wu et al., 2012). Lei et al. reported a significant short-term relationship between lead of PM_2.5_ and TNF-α (percent change = 65.20%, 95% CI: 37.07%, 99.10%) (Lei et al., 2019). A meta-analysis of European cohorts reported a significant long-term association between fibrinogen and zinc of PM_2.5_ (percent change = 1.2%, 95%CI: 0.1%, 2.4%), but an insignificant association for PM_2.5_ mass (Hampel et al., 2015). A review reported that metals in particulate matter play different roles in prothrombotic status (Signorelli et al., 2019). These findings suggest that particles mass alone can’t fully reflect the toxicity of particles. The components of particle contribute to the changes of inflammation and blood coagulation markers with PM exposure. For example, black carbon is reported that more reflected adverse health effect of particulate air pollution compared with PM mass (Janssen et al., 2011). Fang et al. reported that an increase of 37.4% (95%CI: 2.0%, 85.0%) in IL-6, 19.9% (95%CI: 5.3%, 36.4%) in IL-8 and 27.8% (95%CI: 10.0%, 48.4%) in TNF-α per 0.36 μg/m^3^ exposure to black carbon at lag 4 day among patients with diabetes (Fang et al., 2012). Delfino et al. also found black carbon was significant associated with IL-6 in patients with coronary artery disease (Delfino et al., 2008). In an elderly cohort, Zhang et al. reported that IL-6 was significant associated with black carbon, but not with PM_2.5_ (Zhang et al., 2016). Future research should be conducted to investigate associations of PM constituents with inflammation and blood coagulation markers, which can accurately assess the impacts of particulate air pollution on these markers (Pedersen et al., 2016).

Studies used different exposure assessment techniques such as land-use regression, kriging interpolation, and air dispersion modeling. Each exposure assessment technique has its advantages and disadvantages (Beelen et al., 2010). Differences in the accuracy of exposure assessment techniques may contribute to heterogeneities between studies (Sellier et al., 2014). Two studies investigated the long-term association for PM_2.5_-fibrinogen on the same population (Hoffmann et al., 2009; Viehmann et al., 2015). The study modeling PM concentrations on a grid of 5 km reported a significant change of fibrinogen with PM_2.5_ exposure (percent change = 3.9%, 95%CI: 0.3%, 7.7%) (Hoffmann et al., 2009). However, the other study modeling PM concentrations on a grid of 1 km reported an insignificant change of fibrinogen (Viehmann et al., 2015).

Our study had some limitations. First, few studies investigated long-term associations of PM with TNF-α, IL-6, and IL-8. Also, there are few reports on long-term associations of fibrinogen with PM_2.5_ and PM_10_, which leads to lower statistical power. Second, most studies used a single-pollutant model, although there may be interactions between pollutants (Mustafic et al., 2012). It is difficult to implement and validate the multi-pollutant model. Therefore, we estimated the association between inflammation and blood coagulation markers and each pollutant based on the single-pollutant model and did not evaluate the interactions between the air pollutants. Third, significant heterogeneity may come from study design, study location, exposure assessment technique, and population characteristics.

## 5 Conclusion

Our meta-analysis showed fibrinogen and TNF-α were significantly associated with short-term PM exposure. The current study is too limited to draw an appropriate conclusion about long-term associations of PM with the above markers. Future epidemiological studies should address the role long-term PM exposure plays in inflammation and blood coagulation markers level change.

## Declaration of competing interest

The authors declare that they have no known competing financial interests or personal relationships that could have appeared to influence the work reported in this paper.

## Figures and Tables

**figs1:**
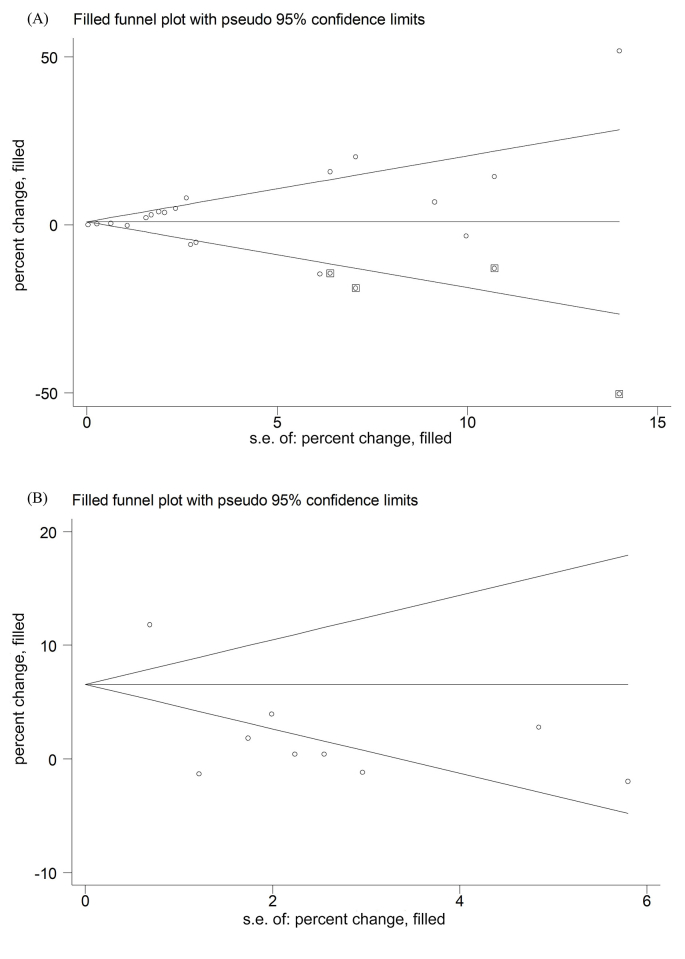


## Footnotes

Appendix A Supplementary data related to this article can be found at https://doi.org/10.1016/j.envpol.2020.115630.
