# Supplementary material for: The short- and long-term associations of particulate matter with inflammation and blood coagulation markers: A meta-analysis
Source: Environ Pollut. 2020 Dec;267:115630. doi: 10.1016/j.envpol.2020.115630 (PMC7687019; doi:10.1016/j.envpol.2020.115630)
Supplement: The following are the supplementary data related to this article:Multimedia component 1 [file mmc1.doc]

**Supplemental Materials**

The short- and long-term associations of particulate matter with inflammation and blood coagulation markers: A systematic review and meta-analysis

Hong Tang^a,b^, Zilu Cheng^c^, Na Li^a,b^, Shuyuan Mao^a,b^, Runxue Ma^a^, Haijun He^a^, Zhiping Niu^a,b^, Xiaolu Chen^a,b^, Hao Xiang^a,b *^

^a^ Department of Global Health, School of Health Sciences, Wuhan University, 115# Donghu Road, Wuhan, China

^b^ Global Health Institute, Wuhan University, 115# Donghu Road, Wuhan, China

^c^ School of Chemistry, Chemical Engineering and Life Sciences, Wuhan University of Technology, 122# Luoshi Road, Wuhan, China

**Table of Contents**

**Supplemental Methods**

Search strings for PubMed

**Tables**

Table S1: Population-Exposure-Comparator-Outcome-Study Design (PECOS) statement

Table S2: Criteria for the risk of bias evaluation of each study, adapted from the Office of Health Assessment and Translation (OHAT) tool

Table S3: Characteristics of studies on the associations of PM with inflammation and blood coagulation markers

Table S4: Risk of bias assessment for each study

**Figure**

Figure S1: Filled funnel plots of “trim-and-fill” analyses **(s.e.: standard error)**

**PRISMA Checklist**

**Supplemental Methods**

**Search strings for PubMed**

#1 ((((("Air Pollution"[Mesh]) OR "Air Pollutants"[Mesh]) OR Air Environmental Pollutants) OR Environmental Air Pollutants) OR pollution) OR pollutant*

#2 ((((((((("Particulate Matter"[Mesh]) OR Airborne Particulate Matter) OR Particulate Air Pollutants) OR Ambient Particulate Matter) OR particulate matters) OR particulate*) OR particle*) OR PM) OR PM2.5) OR PM10

#3 #1 OR #2

#4 (((((("Tumor Necrosis Factor-alpha"[Mesh]) OR Tumor Necrosis Factor alpha[Title/Abstract]) OR Tumor Necrosis Factor Ligand Superfamily Member 2[Title/Abstract]) OR Tumor Necrosis Factor[Title/Abstract]) OR TNF Superfamily, Member 2[Title/Abstract]) OR TNFalpha[Title/Abstract]) OR TNF-alpha[Title/Abstract]

#5 ((((((("Interleukin-6"[Mesh]) OR Interleukin 6[Title/Abstract]) OR IL6[Title/Abstract]) OR IL-6[Title/Abstract]) OR B-Cell Stimulatory Factor 2[Title/Abstract]) OR B-Cell Stimulatory Factor-2[Title/Abstract]) OR BSF-2[Title/Abstract]) OR Hybridoma Growth Factor[Title/Abstract]

#6 ((((((((((("Interleukin-8"[Mesh]) OR Interleukin 8[Title/Abstract]) OR IL8[Title/Abstract]) OR IL-8[Title/Abstract]) OR Monocyte-Derived Neutrophil Chemotactic Factor[Title/Abstract]) OR Neutrophil Activation Factor[Title/Abstract]) OR Chemokine CXCL8[Title/Abstract]) OR CXCL8 Chemokines[Title/Abstract]) OR CXCL8 Chemokine[Title/Abstract]) OR Neutrophil Chemotactic Factor[Title/Abstract]) OR Granulocyte Chemotactic Peptide-Interleukin-8[Title/Abstract]) OR Granulocyte Chemotactic Peptide Interleukin 8[Title/Abstract]

#7 (("Fibrinogen"[Mesh]) OR Blood Coagulation Factor I[Title/Abstract]) OR Coagulation Factor I[Title/Abstract]

#8 #3 AND #4

#9 #3 AND #5

#10 #3 AND #6

#11 #3 AND #7

#12 #8 OR #9 OR #10 OR #11

Table S1 Population-Exposure-Comparator-Outcome-Study Design (PECOS) statement

| Population | Humans (general population, patients), exclude pregnant women |
| --- | --- |
| Exposure | Short-term and long-term exposure to PM_2.5_ (particulate matter with aerodynamic diameter equal to or less than 2.5µm);  Short-term and long-term exposure to PM_10_ (particulate matter with aerodynamic diameter equal to or less than 10µm); |
| Comparator(s)/Control | Not applicable |
| Outcomes | Inflammation and blood coagulation markers: tumor necrosis factor-α(TNF-α), Interleukin 6 (IL-6), Interleukin 8 (IL-8) and fibrinogen |
| Study Design | Epidemiological studies |

Table S2 Criteria for the risk of bias evaluation of each study, adapted from the Office of Health Assessment and Translation (OHAT) tool

| **Bias** | **Risk of Bias Question** | **Answer** |
| --- | --- | --- |
| **Confounding** | Did the study design or analysis account for important confounding and modifying variables? | (1)low risk: All important confounders are considered in the study. For example: age, sex, BMI, temperature, season and day of the week  (2)probably low risk: Most of confounders are considered in the study.  (3)probably high risk: Some but not all of confounders are considered in the study.  (4)high risk: No confounders are considered in the study.  (5)not applicable: Assessment of outcome cannot introduce bias in the study. |
| **Exposure assessment** | Was monitoring technique appropriate for sample population? Includes measurement error or measurement limitations.  List of major considerations: 1) air pollution measurements were performed daily, 2) < 25% missing data, 3) more than one monitoring station per a large geographical area | (1)low risk: Measurement of PM is close to the true average population exposure.  (2)probably low: Indirect evidence suggests low risk of bias, or one of the three listed considerations is not applied.  (3) probably high risk: Indirect evidence suggests high risk of bias. Additionally, two out of the three listed considerations are not applied.  (4) high risk: Direct evidence suggests high risk of bias, or all three of the listed considerations are not applied.  (5) not applicable: Exposure assessment methods are not expected to introduce bias in the study. |
| **Selection bias** | Was sampling technique clearly described, including subject attrition? Were they appropriate for the type of study conducted? Does the selection of participants into the study was done in a manner that might introduce bias in the study? e.g. study only certain days (for examples, only Fridays), and not all days, seasons were included. | (1) low risk: The descriptions of the studied population were sufficiently detailed to support low selection bias.  (2) probably low risk: Indirect evidence suggests low risk of bias of population selection. (3) probably high risk: Indirect evidence suggests high risk of bias of population selection. (4) high risk: Descriptions of the studied population indicated high risk of bias.  (5) not applicable: Selection cannot introduce risk of bias in the study. |
| **Detection bias** | Can we be confident in the outcome assessment? | (1) low risk: Outcome was measured using laboratory testing by professionals.  (2) probably low: Outcome was measured using laboratory testing by researcher.  (3) probably high risk: Outcome was not assessed based on laboratory testing  (4) high risk: Outcome was assessed based on self-reports (parents, family) and data collected by the researcher. |
| **Disease misclassification** | Was disease status confirmed by laboratory test or doctor? This question pertained to studies of patient populations. | (1) low risk: Disease was classified based on physician diagnosis or objective measure (e.g. laboratory test).  (2) high risk: Defined patient populations through self-reports |
| **Selective reporting** | Were all outcomes and findings reported? | (1) low risk: All of the studies pre-specified outcomes and findings are reported  (2) probably low: Indirect evidence suggests study was free of selective report  (3) probably high risk: Indirect evidence suggests study was not free of selective reporting  (4) high risk: Not all pre-specified outcomes and findings were reported  (5) not applicable: Selective outcome reporting cannot introduce bias in the study |

Table S3. Characteristics of studies on the associations of PM with inflammation and blood coagulation markers

| Author year | population | Age  (year)^a^ | sample size | study design | Area | Time | Outcome^a^ | | | | PM level (μg/m^3^)^a^ | | Assessment of exposure to air pollution |
| --- | --- | --- | --- | --- | --- | --- | --- | --- | --- | --- | --- | --- | --- |
|  |  |  |  |  |  |  | TNF-α level (pg/ml) | IL-6 level (pg/ml) | IL-8 level (pg/ml) | fibrinogen level (mg/dL) | PM_2.5_ | PM_10_ |  |
| Payam Dadvand  2014 | COPD patients | 67.8±8.6 | 242 | cross-  sectional | Barcelona, Spain | 01/  2004-  03/  2006 | 0.9±1.4 | 1.4±1.3 | 5.3±3.8 | 420±120* | annual average:  15.8 (3.0) |  | LUR |
| Alexandra Schneider  2010 | diabetic individual | 61±8 | 22 | panel study | USA | 2004-  2005 | 1.7±0.9 | 3.4±2.2 |  |  | 14.3 ± 7.5 |  | air pollution sampler located on the EPHD rooftop, monitoring station |
| Cuicui Wang  2017 | general population | 24 | 36 | panel study | Shanghai, China | 2014-  2015 | 4.22 ±3.61 | 0.33 ±0.16 |  |  | Lag 0-24:  42.00 ±10.70 |  | low-noise personal exposure monitor |
| Sara D. Dubowsky  2006 | general population | ＞60 | 44 | panel study | USA | 03/  2002-  06/  2002 |  | 2.9 (0.98-  18) |  |  | 16 ± 6.0 |  | DustTrak 8520 aerosol monitor installed on portable carts |
| Xian Zhang  2017 | general population | 74.8 ± 7.5 | 97 | panel study | USA | 2012-  2014 |  | 2.4 ± 1.7 |  |  | 17.6 ±7.7 |  | central air monitoring stations |
| Ralph J. Delfino  2010 | subjects with a confirmed history of coronary artery disease | 84.1 ±5.6 | 60 | panel study | USA | 2005-  2007 |  | 2.42 ±1.85 |  |  | warm season: 24.0 ±8.36; cool season: 20.6 ±15.6 |  | outdoor air sampling at each retirement community |
| Rima Habre,  2019 | adults with asthma | 27±9.5 | 22 | randomized crossover study | USA | 2014-  2015 |  | 1.7±2.8 |  | 78.4±29.6 | 12±7.6 | 30.1±22.1 | fixed site |
| Dai-Hua Tsai  2012 | general population | <55: 56.7% | 6183 | cross-  sectional study | Switzerland | 2003-  2006 | 2.87  (1.79-  4.50) | 1.32  (0.58-  3.21) |  |  |  |  | monitoring station located in Lausanne |
| Christie A. Cole  2018 | general population | 29 ± 5.6 | 38 | randomized crossover study | Canada | 2010-  2011 |  | 3.6 ±4.3 |  |  | 7.3 ±5.3 | 13±7.3 | A GRIMM Dust Monitor placed into a separate rear pannier |
| Shaowei Wu  2012 | general population | <60 | 40 | panel study | China Beijing | 2008-  2010 | 0.62-  7.88 |  |  |  | 57.4  (63.4) | 112.0  (73.5) | a central air-monitoring station located within 300 meters of school dormitory |
| Mohammad Sadegh Hassanvand  2017 | general population | 16.2 ± 0.5 | 40 | panel study | Tehran | 2012-  2013 |  | 15.61 ± 26.32 |  |  | 44.1 ±9.7 |  | PM monitoring stations |
| Renjie Chen  2018 | general population | 20 | 60 | double-  blind, randomized crossover study | Shanghai, China | 11/  2015-  12/  2015 | Sham-  purified air: 57; True-  purified air: 50 | Sham-  purified air:88; True-  purified air: 81 |  |  | 53.1±9.4 |  | monitored at the rooftop of the campus and 13 fixed-site monitoring stations |
| Premkumari Kumarathasan  2018 | general population | 18–34 | 52 | randomized cross-over study | Canada | 2010 |  |  |  |  | 12.23 ±6.80 |  | a fixed site ambient air quality monitor |
| C. Arden Pope III  2016 | general population | 23 ±2 | 72 | panel study | USA | 2013-  2015 | 20.79±  18.91 | 2.45±4.92 | 42.42±68.69 |  |  |  | three monitoring sites |
| Jaime E. Mirowsky  2015 | general population | 25 | 23 | randomized crossover study | USA | 2011-  2012 |  |  | SF: 3.3; GSP:2.4; GWB:  2.0 |  | 20 | 26 | a custom-  built mobile sampling platform |
| Kai-Jen Chuang  2007 | healthy college students | 20.8±1.2 | 76 | panel study | Taiwan, China | 04/  2004-  06/  2005 |  |  |  | 296.6±  54.2 | 3-day average：36.5±  12.6 | 1-day average  49.2±  18.0； | PM supersite monitoring station, located 1 km from the Fu-Jen campus |
| Daniel P. Crofta  2017 | cardiac patients | 30-89 | 135 | cross-  sectional | USA | 01/11/2011-  31/12/2013 |  |  |  | 181.3±  65.2** | 24h average: 6.9±3.1 |  | collected at the New York State DEC site in Rochester |
| J. Emmerechts  2011 | patients with diabetes | 57.9±17.5 | 233 | panel study | Leuven, Belgium | 02/  2010-  04/  2010 |  |  |  | 340±80* |  | mean 1 week：25.8  (16.5) | kriging interpolation method |
| Lindsay J. L. Forbes  2009 | general population | ≥16 | 25,000 | cross-  sectional | England | 1994；1998；2003 |  |  |  | 1994:1998:2003 Male*:  289;  253;281; Female*:  315;270;  302 |  | annual average 1994:  19.5  (3.7); 1998:  17.9  (2.7); 2003:  16.2  (2.6) | air dispersion models |
| Rochelle Green  2016 | midlife women | 46.3±2.7 | 2086 | panel study | USA | 1999-  2004 |  |  |  | 286.4±  58.1 | 30-day average:  15.9±5.5 1-year average:  16.4±3.4 |  | monitors located within their 20 km buffers |
| Anjum Hajata  2015 | general population | 62±10 | 6814 | panel study | USA | 2000-  2012 |  | 1.6 ±1.2 |  | 348.8±  74.9 | annual average  16.5±3.4； lag 0: 17.2±10.2; |  | central site monitor in each region |
| Katharina Hildebrandt  2009 | patients with chronic pulmonary disease | 53.8 ±12.3 | 38 | panel study | Erfurt, Germany | 15/10/2001-  6/5/  2002 |  |  |  | 310±70* |  | 17.73±  10.29 | a fixed monitoring station |
| Barbara Hoffmann  2009 | general population | 45–75 | 4032 | cross-  sectional | Germany | 2000-  2003 |  |  |  | Men:  317 (95);  Women: 332(98) | annual mean:  22.8 | daily mean:  37.7±  19.7 | EURAD model |
| Kati Huttunen  2012 | ischemic heart disease patients | 71.2 | 52 | panel study | Kotka, Finland | 11/  2005-5/2006 |  |  | 3.1±2.4 | 310±60* | daily average:  8.7±5.1 |  | a fixed outdoor air pollution monitoring site |
| Joel Schwartz  2001 | general population | 49±20 | 20000 | cross-  sectional | USA | 1989-  1994 |  |  |  | 318±89 |  | 35.2±  20.5 | weighted average of all monitors in their county of residence |
| Timo Lanki  2015 | general population | KORA  50.2； HNR  59.5；FINRISK：48.9； 60-year-old:60.4 | 17,428 | cross-  sectional | European | 1994-  2007 |  |  |  | KORA：276±67*；HNR：330±80*;  FINRISK:  355±81*;  60-year-  old：302±77* | annual mean:  KORA：13.6；HNR：18.4；FINRISK: 7.7；60-year-  old: 7.3 | annual mean:  KORA  20.3；  HNR：27.8；FINRISK14.1；60-year-  old: 15.0 | Land use regression models |
| Hyewon Lee  2018 | general population | <65:  89.9%  ≥65:  11.1% | 6589 | panel study | Seoul, South Korea | 2010- 2016 |  |  |  | 149-525 | 8-day average：24.5±8.7annual average：24.5±2.0 | 8-day average  46.1±  18.4； annual average  45.8±  3.7 | each district has a centrally located regular monitoring site |
| Duanping Liao  2005 | middle-  age males and females | 54±5.8 | 10208 | cross-  sectional | USA | 1987-  1989 |  |  |  | 302.1±  65.0 |  | Lag1：29.9±12.8 | monitor-specific measures |
| J Pekkanen  2000 | male and female office workers | 35–55 | 4042 | cross- sectional | London | 09/  1991-  05/  1993 |  |  |  | 242* |  | 31.4 | measured in central London，from five sites |
| David Q. Rich  2012 | patients undergoing Cardiac Rehabilitation | 17%<50；3% >80 | 76 | panel study | USA | 06/2006-11  /2009 |  |  |  | 358±87* | daily  8.67±6.06 |  | a TEOM at the NYS DEC site in Rochester |
| Regina Rückerl  2007 | myocardial infarction survivors | Helsinki:  64.6;  Stockholm:  64.0;  Augsburg:  61.9;  Rome:62.7;  Barcelona:  62.1;  Athens:  54.7 | 1003 | panel study | Europe | 05/  2003-  07/  2004 |  | Helsinki: 3.16; Stockholm2.67; Augsburg: 2.60; Rome: 3.18; Barcelona: 3.58; Athens: 3.19 |  | Helsinki:  376*; Stockholm:353*;  Augsburg:334*;  Rome:  324*;  Barcelona:399* | 24 h averages Helsinki:  8.2; Stockholm8.8; Augsburg:17.4; Rome:  24.5; Barcelona:24.2 | 24 h average Helsinki:  17.1; Stockholm  17.8; Augsburg:33.1; Rome:  42.1; Barcelona:  40.7; | central monitoring sites in each city |
| Regina Rückerl  2014 | Gen.susc.;  T2D; IGT | All:  63.1±10.9；  T2D or IGT：  66.4±8.5；  Gen.susc.：55.9±12.1 | Gen.susc:87;  T2D:83;  IGT:104 | panel study | Augsburg, Germany | 19/03/2007-  17/12/2008 |  | All: 1.4±3.1; T2D or IGT: 1.7±3.7; Gen.susc1.0±0.9 |  | All:  360±60*;  T2D or IGT：370±70*;  Gen.susc.:  330±50* | 24h averages  13.7±10.0 | 24h average  18.3±12.0 | a fixed monitoring site throughout the study period |
| Goran Rudež  2009 | healthy volunteers | 41±15 | 40 | panel study | RotterdamNetherlands | 01/  2005-  12/  2006 |  |  |  | 260±50* |  | 24h mean:  29.3 (23.8, 39.2) | monitoring station |
| Anthony Seaton  1999 | subjects aged over 60 years | Belfast  70.4±6.45；Edinburgh：68.2±5.5 | 108 | panel study | Belfast and Edinburgh UK | 1/11/  1996 - 30/04/1998 |  |  |  | NA |  | Belfast  42±2.0  Edinburgh  57±1.78； | fixed site samplers in each city, measured by TEOM |
| Arie Steinvil  2008 | general population | males: 46±12; females:  46±11 | Total:  3659; males:  2203; female:  1456 | cross-  sectional | Tel-Aviv, Israel | 09/  2002-  07/  2006 |  |  |  | males：280±60；females：304±58 |  | daily average  64.5±  100.8 | air pollution monitoring stations located at roof |
| Maciej Strak  2013 | healthy adult | 22 | 31 | semi-  experimental design | Netherland | 03/  2009-  10/  2009 |  |  |  | 302* | 39 | 76 | measured with Harvard impactors at selected sites |
| Ta-Chen Su  2017 | middle-  aged adults | 47.6 ± 6.8 | 402 | cohort study | Taiwan, China | 2009-  2011 |  |  |  | 265.42 ±  59.89 | annual average:  29.08 ±  5.10 | annual average  47.82 ±   4.78 | LUR  model |
| Jeffrey H Sullivan  2007 | healthy elderly individual;  COPD patients;  CVD patients | healthy： 66-88；COPD:  65-89；CVD： 56-86 | Healthy:8  COPD:16  CVD:23;  Total:  47 | Panel study | Seattle, USA | 02/  2000-  05/  2001；12/  2001-  03/  2002 |  |  |  | Healthy: 313-511;  COPD:  327-617;  CVD: 268-674 | 24-hour average: 7.7; |  | measured by nephelometry outside of the participant's residence |
| Ariana Zeka  2006 | general population | 73.0±6.7 | 710 | cross-sectional | Greater Boston area，USA | 14/11/2000-  31/12/2004 |  |  |  | 348.5±  88.5 | lag 2 day  11.16±  7.95 |  | Harvard School of Public Health monitoring sites |
| Moniek Zuurbier  2011 | healthy adults | 42.0 | 34 | semi-experimental design | Netherlands | 06/  2007-  06/  2008 | 2.0 ±0.11 | 0.41±  0.023 | 3.0 ±  0.33 | 300* | NA | 28 | PM2.5:active sampling personal;  PM10:beta attenuation continuous monitors |
| Anja Viehmann  2015 | general population | 45-74 | 4814 | cohort study | Germany | 2000-  2003；2006-  2008 |  |  |  | 323 (95) | 365-day average： 16.7±1.6  1-3 days average： 16.9±8.3 | 365-day average  20.7±2.5；  1-3 days average  20.9±9.6 | chemistry transport and dispersion model |
| Robin C. Puett 2019 | patients with diabetes | 12.3±4.4 | 2566 | cross-  sectional | USA | 2002-  2006 |  | 18.4±  24.9 ug/L |  | 352.5±  72.1 | 11.2±5.5 |  | spatio-  temporal models |
| Zhonghua Deng 2020 | patients |  | 1092 | time-series | China | 2014-  2016 |  |  |  | 222.11±  71.31 g/L | 61.91±  39.90 |  | 11 fixed-site station |
| Qingli Zhang  2020 | healthy adults | 24.5±1.5 | 40 | Panel study | China | 05/  2016-  10/  2016 | 7.27±  4.21 | 1.40±  1.35 | 9.51±  7.18 |  | 41.09±  21.13 |  | Fixed-site |

a: Data were extracted in the form of "mean ± SD" or "Range" or "median (IQR) " or "median(25th, 75th)" or "mean"; *1 g/L=100 mg/dL; **1 ug/mL=10^-1^ mg/dL.

Abbreviations: TNF-α: tumor necrosis factor α; IL-6: interleukin 6; IL-8: interleukin 8; CVD: cardiovascular disease; COPD: chronic obstructive pulmonary disease; EURAD model: European Air Pollution Dispersion model; IGT: impaired glucose tolerance; T2D: type 2 diabetes mellitus; Gen. susc.=genetically susceptible subjects; TEOM: Tapered element oscillating microbalance; LUR=land use regression; GWB=George Washington Bridge, GSP=Garden State Parkway, SF=Sterling Forest, DEC=Department of Environmental Conservation, KORA =Cooperative Health Research in the Region of Augsburg, HNR =Heinz Nixdorf Recall

Table S4 Risk of bias assessment for each study

| **Risk of bias: Epidemiologic Studies** | **Selection bias** | **Disease misclassification** | **Exposure assessment** | **Confounding** | **Detection bias** | **Selective reporting** |
| --- | --- | --- | --- | --- | --- | --- |
| Short-term exposure to PM_2.5_ | | | | | | |
| Chen 2018 | probably low | N/A | probably low | low | probably low | low |
|  | 60 healthy students from 17 dormitory rooms at the Jiangwan campus of Fudan University. |  | PM_2.5_ measurements (per half-hour) monitored at the rooftop of building as surrogates of the exposure levels, hourly PM_2.5_ data from 13 fixed-site monitoring stations for occasional exposure outside the campus | age, gender, body mass index, and daily average temperature and relative humidity | ELISAs | yes |
| Chuang 2007 | probably low | **N/A** | probably low | low | low | low |
|  | 76 healthy college students(ages 18-25) recruited from university; no history of smoking; no medication that might affect cardiac rhythm; and no cardiovascular diseases |  | hourly concentrations of PM_10_ and PM_2.5_ from the air-monitoring station in the Fu-Jen campus | sex, age, BMI, weekday, temperature of the day before, relative humidity | clotting method of Clauss, using STA-Fibrinogen 5 kits | yes |
| Cole 2018 | low | **N/A** | low | probably low | low | low |
|  | healthy adult (ages 19–39) participants, recruited using advertisements |  | A GRIMM Dust Monitor placed into a separate rear pannier measured PM_2.5_ and PM_10_ at 6-s intervals | BMI, age and sex variables with the Route variable scaled to the Residential route | ELISA Human IL-6 Immunoassay | yes |
| Crofta 2017 | probably low | probably low | probably low | low | low | low |
|  | patients (>18) with either acute coronary syndrome or a non-emergent cardiac catheterization for stable SIHD, excluded patients with unstable angina | treated at the Cardiac Catheterization Laboratory | a central site monitor, measured variables were averaged to 1 h values | age, dyslipidemia, prior MI, smoking, year, weekday, hour of the day, temperature, relative humidity | ELISA by Assay Gate | yes |
| Dadvand 2014 | probably low | low | probably low | low | low | low |
|  | clinically stable COPD patients | the American Thoracic Society/European Respiratory Society definition | LUR models for PM_2.5_, at the home address during 10 windows of exposure | age, sex, education level, BMI and recruiting hospital | using the Clauss method as part of routine blood exams | yes (full range of lag periods analyzed presented) |
| Delfino 2010 | probably low | low | low | probably low | low | low |
|  | ≥65, with a confirmed history of coronary artery disease, nonsmoker, without ETS exposure | a confirmed history of coronary artery disease | outdoor air sampling at each retirement community | temperature, seasonal phase, medications, asthma | 96-well immunoassay kits | yes |
| Dubowsky 2006 | low | **N/A** | low | low | low | low |
|  | 44 nonsmoking seniors, in suburban St. Louis, individuals with atrial flutter, atrial fibrillation, and/or a paced rhythm were excluded |  | ambient PM data were obtained from the U.S. Environmental Protection Agency, group-level PM_2.5_ using DustTrak 8520 aerosol monitor installed on portable carts | sex, obesity, diabetes, smoking history (ever/never), residence, mold, pollen, illness, juice intake, temperature | the Clinical and Epidemiologic Research Laboratory at Boston Children’s Hospital, using ELISAs | yes |
| Green 2016 | probably low | **N/A** | probably low | low | low | low |
|  | women through the menopausal transition(42 - 52), having an intact uterus and one or more ovaries, not being pregnant or lactating, not using reproductive hormones in the past 3 months |  | multiple monitors, with varying dates in service and completeness of data, were located within 20 km of a woman's residence. PM_2.5_ was typically measured every three days, but sometimes every six days or daily. | study site, race/ethnicity, education, age, active smoking, BMI, alcohol consumption in the 24 hours prior to the blood draw | a turbidometric detection system | yes |
| Hajata 2015 | low | **N/A** | low | low | low | low |
|  | participants from the Multi-Ethnic Study of Atherosclerosis |  | regulatory monitoring stations from the US Environmental Protection Agency’s AQS, monitors deployed by MESA Air at fixed sites, outdoor monitors at participant’s homes, and monitors placed to better capture roadway concentration gradients | age, race, gender, education, income, employment status, neighborhood SES, smoking status, secondhand smoke exposure, current alcohol consumption, BMI, waist–hip ratio, diabetes, hypertension, use of medications | fibrinogen antigen assays were performed using BNII nephelometers | yes, a priori exposures (day prior, concurrent, 2day, 3 day, 4day, 5day averages) presented |
| Hassanvand 2017 | probably low | **N/A** | low | probably low | low | low |
|  | healthy young adults, located in central urban area of Tehran |  | twenty-four-hour PM sampling was conducted in each sampling site, real-time data collected by the GRIMM dust monitors | temperature, relative humidity | ELISAs  at Immunology, Asthma and Allergy Research Institute, | yes |
| Huttunen 2012 | low | low | probably low | probably high | low | low |
|  | elderly ischemic heart disease patients, from Kymenlaakso Central Hospital and Health Care Center of Kotka, non-smoker, without chronic inflammatory diseases | diagnosed by a physician (diagnoses I20–I25 according to the 10^th^ revision of the International Classification of Diseases) | a fixed outdoor air pollution monitoring site, within two kilometers of residence | time-trend, apparent temperature | analyzed in the Kymenlaakso Hospital Services, a chromogenic method | yes, all lag periods and outcomes reported |
| Kumarathasan 2018 | low | **N/A** | probably low | low | low | low |
|  | healthy, college students (18-34), did not use medications, did not have a history of chronic disease, non-smoking, without cigarette smoke exposure at home |  | a fixed site ambient air quality monitor, analyses of PM_2.5_ by nephelometry | age, sex, BMI, ambient air pressure, humidity, temperature | affinity-based multiplex protein array assays using Bio-Plex Pro Human panels and Milliplex Map kits with a Bioplex 100 instrument | yes |
| Lee 2018 | low | **N/A** | probably low | low | low | low |
|  | from a cohort of 84,914 subjects who underwent health check-ups at the Samsung Medical Center, non-smokers or ex-smokers, >3 hospital visits, residents of one of the 25 districts of Seoul |  | hourly PM_2.5_,PM_10_: a centrally located regular monitoring site at each district | age, sex, BMI, morbidity, the proportion of green space, temperature, relative humidity | a clotting assay with STA-Fibrinogen and STA-Owren Koller Buffer | yes (full range of lag periods analyzed presented ) |
| Mirowsky 2015 | low | **N/A** | low | probably low | low | low |
|  | healthy adult(18–40), from the Northern New Jersey, via personal contacts and flyers, excluded tobacco use |  | collected PM using a custom-built mobile sampling platform | ambient temperature, relative humidity, location, random subject effects | using Meso Scale Discovery | yes |
| Pope III 2016 | low | **N/A** | low | probably low | low | low |
|  | healthy, young adults recruited from Provo, nonsmokers, without exposure to second-hand smoke |  | daily ambient concentrations of PM_2.5_ from 3 monitoring sites, | sex, fish oil supplement use, time exercising, menstruation | by analytic services at Eve Technologies | yes |
| Rich 2012 | low | low | probably low | probably low | low | low |
|  | patients participated in cardiac rehabilitation program. excluded participants with cardio­myopathy, coronary bypass grafting within the last 3 months | their cardiologist to the University of Rochester Cardiac Rehabilitation Center | a tapered element oscillating microbalance at the NYS DEC site in Rochester | temperature, barometric pressure, relative humidity, sulfur dioxide, carbon monoxide and ozone | measured in the Strong Memorial Hospital Clinical Laboratories | yes (lag hr 0–5, lag hr 24–47, 48–71, 72–05, 96–119) |
| Rückerl 2007 | low | probably low | probably low | low | low | low |
|  | MI patients (35–80 years )was performed in six European cities, without MI or interventional procedures < 3 months before the beginning of the study | MI patients but case confirmation not explicitly stated. | through city-specific air monitoring networks | age, sex, and BMI, time-varying confounders, active smoking | measured by a fully automated assay | yes(lag0, lag1, lag2 and 5-day average) |
| Rückerl 2014 | low | low | probably low | low | low | low |
|  | individuals with T2D, IGT, a potential genetic predisposition on detoxifying pathways, from a large ongoing study, non-smokers or ex-smokers for at least twelve months | OGTT | hourly means of PM_2.5_ were measured at a fixed monitoring site | sex, intake of medication, BMI, age, SNP rs1205 located on the CRPgene on hs-CRP levels and of the SNP rs18000790 located on the fibrinogen gene on fibrinogen levels | immunonephelometry | yes(lag0,  lag1,lag2,  lag3,lag4 and 5-day average) |
| Habre 2019 | probably low | low | low | low | low | low |
|  | 22 adults(≥18),with mild to moderate asthma, non-current smokers, advertising to USC staff and students | defined by symptoms-based NHLBI criteria | a mobile monitoring platform at each park in a stationary location to obtain more detailed characterization | asthma control, allergic status, race and ethnicity, physical activity levels, BMI, commuting patterns | Interleukin 6: ELISA kits, fibrinogen: the Millipore Luminex magnetic bead panel | yes |
| Schneider 2010 | probably low | low | low | low | low | low |
|  | 22 volunteers(48-78), with type 2 diabetes, from UNC Diabetes Clinic | have a diagnosis of type 2 diabetes, identified through the UNC Diabetes Clinic | a 3000K Versatile Air Pollution Sampler; daily 24-hour PM_2.5_ were obtained from a monitoring station | BMI, age, HbA1c, adiponectin, MPO, ferritin, medication intake, GSTM1 null polymorphism | commercially available ELISA kits and run on a Luminex 100 Multiplex system | yes |
| Strak 2013 | probably low | **N/A** | low | low | low | probably low |
|  | healthy, young, non-smoking students of Utrecht University |  | The PM_2.5_ mass concentrations were measured with HI operating | temperature, relative humidity, season, use of oral contraceptives, | a BCS with Multifibren U kit |  |
| Sullivan 2007 | probably low | low | low | low | low | low |
|  | recruited by advertisement in senior centers, local newspapers, medical clinics. CVD, COPD and healthy participants (≥55), non-smokers | physician diagnosed | PM was measured by nephelometry located within 2-miles of the subjects' residence | age, gender, temperature, relative humidity, anti-inflammatory medication use and statin use | fibrinogen levels were measured in citrated plasma by the Clauss method | yes (all lag periods reported: Lag0 and Lag 1) |
| Wang 2017 | probably low | **N/A** | low | low | low | low |
|  | 36 healthy college students, in the School of Public Health, Fudan University |  | the MicroPEM, record PM_2.5_ concentration every 10.0 seconds | age, sex, BMI, time-varying temperature, humidity, day of the week, season | TNF-α:the Millipore MILLIPLEX MAP human cytokine/chemokine kit; IL-6: ELISAs | yes |
| Wu 2012 | probably low | **N/A** | probably low | low | low | low |
|  | male, healthy college students, in Beijing, recruited from a local university, in good health, free of any cardiovascular, pulmonary or other chronic diseases |  | PM_2.5_ in a central air-monitoring station located within 300 meters of school dormitories, PM_10_ from the nearest governmental air-monitoring stations | age, BMI, time trend, day-of-week, study location, temperature and relative humidity | ELISAs  following standard methods | yes |
| Zeka 2006 | low | **N/A** | probably low | low | low | low |
|  | 710 currently active subjects (21–80) from NAS, free of known chronic medical conditions |  | measured at the Harvard School of Public Health monitoring sites | age, BMI, season, temperature, relative humidity, barometric pressure, use of medications, the presence of hypertension, smoking, alcohol drinking, fasting glucose levels | fibrinogen: Fibriquick method fibrinogen | yes  (moving averages for PM_2.5_ concentration for the 48h, 1 and 4 weeks) |
| Zhang 2017 | probably low | **N/A** | probably low | low | low | low |
|  | 97 elderly non-smoking adults living in two Los Angeles California metropolitan areas |  | central air monitoring stations, hourly PM_2.5_ from Beta attenuation monitors | age, sex, obesity, history of hypertension, hypercholesterolemia, intake of medication, diabetes mellitus, total cholesterol/HDL ratio, cardiovascular risk score, intake of statin, study area, former smokers | 96-well immunoassay kits | yes |
| Zuurbier 2011 | probably low | **N/A** | low | low | low | low |
|  | 34 healthy, non-smoking volunteers(18-56), selected through intranet web sites of their employers |  | measured PM_2.5_ with active sampling personal DataRAMs | relative humidity, temperature, cycling versus riding by car/bus, BMI, fruit and vitamin intake, season, minutes of traffic participation before baseline measurements | The STA‑R automated coagulation analyzer | yes |
| Long-term exposure to PM_2.5_ | | | | | | |
| Dadvand 2014 | probably low | low | probably low | low | low | low |
|  | clinically stable COPD patients | diagnosis of COPD according to the American Thoracic Society/European Respiratory Society definition | at the home address by LUR models | age, sex, education level, BMI and recruiting hospital | using the Clauss method as part of routine blood exams | yes |
| Green 2016 | probably low | **N/A** | probably low | low | low | low |
|  | women through the menopausal transition(42 - 52), having an intact uterus and one or more ovaries, not being pregnant or lactating, not using reproductive hormones and had at least one menstrual period in the past 3 months |  | multiple monitors, were located within 20 km of a woman's residence | age, marital status, income, education, BMI, smoking, alcohol use, hormone therapy, statin use, menopausal stage, hypertension, diabetes | a turbidometric detection system | yes |
| Hajata 2015 | low | **N/A** | low | low | low | low |
|  | participants from the Multi-Ethnic Study of Atherosclerosis |  | regulatory monitoring stations from the US Environmental Protection Agency’s AQS, monitors deployed by MESA Air at fixed sites, outdoor monitors at participant’s homes, and monitors placed to better capture roadway concentration gradients | age, race, gender, education, income, employment status, neighborhood SES, smoking status, secondhand smoke exposure, current alcohol consumption, BMI, waist–hip ratio, diabetes, hypertension, use of medications | fibrinogen antigen assays were performed using BNII nephelometers | yes, a priori exposures (day prior, concurrent, 2day, 3 day, 4day, 5day averages) presented |
| Lanki 2015 | low | **N/A** | low | low | low | **N/A** |
|  | participants from FINRISK, KORA, HNR, and the “60-year-olds” cohort |  | In each study area, pollutants were measured in different locations for 2 weeks during winter, summer, and an intermediate season; annual outdoor concentrations of air pollution were modeled at the participants’ home addresses using LUR model | age, sex, education, BMI, smoking status, physical activity, alcohol intake, neighborhood level | KORA: immunonephelometry on a BN II analyzer; HNR: a Clauss method using an automated BCS-Analyzer; FINRISK: Clauss method using IL Test Fibrinogen-C kit and ACL300R equipment; "60-year-olds": a functional spectrophotometric test |  |
| Lee 2018 | low | **N/A** | probably low | low | low | low |
|  | from a cohort of 84,914 subjects who underwent health check-ups at the Samsung Medical Center, non-smokers or ex-smokers, >3 hospital visits, residents of one of the 25 districts of Seoul |  | each district has a centrally located regular monitoring site to measure PM_2.5_ | age, sex, BMI, morbidity, the proportion of green space, ambient temperature and relative humidity | a clotting assay with STA-Fibrinogen and STA-Owren Koller Buffer | yes |
| Su 2017 | low | **N/A** | probably low | low | low | low |
|  | from a cohort study on work and environment-related cardiovascular diseases as the control subjects of acute CHD in NTUH (35–65) |  | LUR models were used to estimate annual average air pollution concentrations at the participants’ home addresses | age, sex, BMI, systolic BP, LDL-C, lipid-lowering treatment, diabetes mellitus, ever smoking history and hs-CRP | clotting method of Clauss and STA-Fibrinogen Kits | yes |
| Viehmann 2015 | low | **N/A** | probably low | low | low | low |
|  | from prospective cardiovascular cohort study (The German Heinz Nixdorf Recall Study) |  | EURAD chemistry transport and dispersion model, at participant’s home address | age, smoking status, ETS, economic activity, physical activity, BMI, alcohol intake, diabetes, cardiovascular disease, statin intake, season, temperature | an automated BCS-analyser | yes |
| Short-term exposure to PM_10_ | | | | | | |
| Chuang 2007 | probably low | **N/A** | probably low | low | low | low |
|  | 76 healthy college students(18-25); no history of smoking; no medication that might affect cardiac rhythm; and no cardiovascular diseases |  | air-monitoring station in the Fu-Jen campus | sex, age, and BMI, and weekday, temperature of the day before, relative humidity | clotting method of Clauss, using STA-Fibrinogen 5 kits | Yes  (moving averages for 24, 48 and 72 hours) |
| Emmerechts 2011 | low | low | low | low | low | low |
|  | persons with either type 1 or type 2 diabetes(>18 years old), from the diabetes outpatient clinic at the University Hospital Leuven; current non-smokers, not on anticoagulant therapy | physician-  diagnosed and under treatment | current exposure: a portable laser-operated aerosol mass analyzer; subacute, subchronic and chronic exposure: a kriging interpolation method | gender, age, body mass index, socioeconomic status, type of diabetes, physical activity, blood glucose levels, use of medication, temperature, humidity | standard clinical procedures on automated analyzers | yes |
| Hildebrandt 2009 | probably low | low | probably low | low | low | low |
|  | male patients with chronic pulmonary disease, recruited by practitioners or through newspaper advertisement, excluded patients with pacemaker, insulin dependent diabetes mellitus, recent MI, bypass surgery or balloon dilatation less than three months ago | baseline questionnaire on health status and clinical examination | recorded hourly at a fixed monitoring station | long-term time trend, air temperature, relative humidity, barometric pressure, airway infection, medical attendance, hospital admission, corticosteroid or antibiotic intake | immunonephelometry | yes (all lag periods reported: Lag0, Lag 1, Lag2, Lag3, Lag4 and 5 day average) |
| Hoffmann 2009 | low | **N/A** | probably low | low | low | low |
|  | participants from prospective cardiovascular cohort study (the Heinz Nixdorf Recall Study) |  | the EURAD model | age, area of residence, smoking behavior, ETS, BMI, waist circumference, physical activity, alcohol consumption, LDL, HDL | an automated BCS analyzer | yes |
| Lee 2018 | low | **N/A** | probably low | low | low | low |
|  | from a cohort of 84,914 subjects who underwent health check-ups at the Samsung Medical Center, non-smokers or ex-smokers, >3 hospital visits, residents of one of the 25 districts of Seoul |  | each district has a centrally located regular monitoring site to measure hourly concentrations of PM_10_ | age, sex, BMI, morbidity, the proportion of green space, ambient temperature, relative humidity | a clotting assay with STA-Fibrinogen and STA-Owren Koller Buffer | yes (a priori exposures (day of blood draw, 2,3,4,6,8-dday average)) |
| Liao 2005 | low | **N/A** | low | low | low | probably high |
|  | from the Atherosclerosis Risk in Communities (ARIC) study cohort(45–64) |  | PM_10_ data obtained from the AIRS database were monitor-specific daily (24-h) averages | age, sex, ethnicity-center, education, smoking, drinking status, BMI, history of disease, humidity, season, temperature, cloud cover | assays were performed according to standardized protocols | did not report all described lag periods |
| Pekkanen 2000 | probably low | **N/A** | probably low | low | low | low |
|  | employees from 20 London based civil service departments (35–55) |  | 24-h averages of PM_10_, measured in central London | sex, employment grade, ethnicity, smoking, BMI, alcohol consumption, month of examination | an automated modification of the Clauss method | Yes  (reported all described lag periods: lag0,lag1,  lag2,lag3 ) |
| Rückerl 2007 | low | probably low | probably low | low | low | low |
|  | MI patients (35–80)was performed in six European cities, without MI or interventional procedures < 3 months | MI patients but case confirmation not explicitly stated. | through city-specific air monitoring networks | age, sex, and BMI, time-varying confounders, active smoking | measured by a fully automated assay | yes(lag0, lag1, lag2 and 5-day average) |
| Rückerl 2014 | low | low | probably low | low | low | low |
|  | individuals with T2D, IGT, a potential genetic predisposition on detoxifying pathways, from a large ongoing study, non-smokers or ex-smokers for at least twelve months | OGTT | hourly means of PM_10_ were measured at a fixed monitoring site | sex, intake of medication, BMI, age, SNP rs1205 located on the CRP gene on hs-CRP levels,  the SNP rs18000790 located on the fibrinogen gene on fibrinogen levels | immunonephelometry | yes(lag0,  lag1,lag2,  lag3,lag4 and 5-day average) |
| Rudez 2009 | probably low | **N/A** | low | probably low | low | low |
|  | 40 healthy individuals, excluded symptoms of chronic infectious diseases, acute infections, or any surgical procedure within the preceding 3 months |  | from the Dutch National Air Quality Monitoring Network | temperature, pressure, relative humidity | according to von Clauss | yes |
| Habre 2019 | probably low | low | low | low | low | low |
|  | 22 adults, aged 18 years or older with mild to moderate asthma, non-current smokers, advertising to USC staff and students | defined by symptoms-based NHLBI criteria | a mobile monitoring platform at each park in a stationary location to obtain more detailed characterization | asthma control, allergic status, race and ethnicity, physical activity levels, BMI, commuting patterns | Interleukin 6: ELISA kits, fibrinogen: the Millipore Luminex magnetic bead panel | yes |
| Seaton 1999 | probably low | **N/A** | low | probably high | low | low |
|  | Subjects from lists on the basis of a representative distribution around Belfast and Edinburgh(>60), no current smoker |  | UK Department of the Environment, Transport and the Regions’ fixed site samplers in each city, measured by TEOM | minimum temperature, individual city effects | Clauss method | yes |
| Schwartz 2001 | low | **N/A** | low | low | low | low |
|  | subject from NHANES III |  | from AIRS containing information on all of the routine pollution monitoring in the United States. | age, ethnicity, BMI, smoking status, cigarettes per day, sex | laboratory test | yes |
| Steinvil 2008 | low | **N/A** | probably low | low | low | low |
|  | patients attending the Tel-Aviv Sourasky Medical Center, for a routine health examination, without inflammatory disease (arthritis, inflammatory bowel disease, psoriasis, etc.),pregnancy, steroidal or nonsteroidal treatment, acute infection or invasive procedures during the last 6 months |  | air pollution monitoring stations located at roof height in representative areas | age, waist circumference, BMI, LDL, HDL, triglycerides, diastolic and systolic blood pressure, glucose concentration, alcohol consumption, sport intensity, medications, smoking status, history of disease, temperature, relative humidity, precipitation, season | quantified by the method of Clauss and a Sysmex 6000 autoanalyzer | yes (all lag periods reported: Lag0- Lag1 and 7-day average) |
| Strak 2013 | probably low | **N/A** | low | low | low | probably low |
|  | healthy, young, non-smoking students of Utrecht University, living at the campus |  | The PM_10_ mass concentrations were measured with HI operating | temperature, relative humidity, season, use of oral contraceptives, | fibrinogen was measured on a BCS with Multifibren U kit |  |
| Tsai 2012 | low | **N/A** | probably low | low | low | low |
|  | participants to the CoLaus study, aged 35–75 years, selected using a simple, non-stratified random selection approach |  | the monitoring data was obtained from the website of Swiss National Air Pollution Monitoring Network | age, sex, BMI, smoking status, alcohol consumption, diabetes status, hypertension status, education levels, zip code, statin intake, temperature, barometric pressure, season | sent in dry ice to the laboratory, using a multiplexed particle-based flow cytometric cytokine assay | yes |
| Zuurbier 2011 | probably low | **N/A** | low | low | low | low |
|  | 34 healthy, non-smoking volunteers(18-56), selected through intranet web sites of their employers |  | PM_10_ concentrations measured with beta attenuation continuous monitors, from the Dutch National Air Quality Monitoring Network | relative humidity, temperature, cycling versus riding by car/bus, BMI, fruit and vitamin intake, season, minutes of traffic participation before baseline measurements | The STA-R automated coagulation analyzer | yes |
| Long-term exposure to PM_10_ | | | | | | |
| Forbes 2009 | low | **N/A** | probably low | low | low | low |
|  | annual surveys of representative samples of people living in private households in England |  | using air dispersion models | age, sex, BMI, social class of head of household, cigarette smoking, region | 1994: nephelometric method; 1998 and 2003: a modification of the Clauss thrombin clotting method | yes |
| Lanki 2015 | low | **N/A** | low | low | low | **N/A** |
|  | participants from FINRISK, KORA, HNR, and the “60-year-olds” cohort |  | In each study area, pollutants were measured in different locations for 2 weeks during winter, summer, and an intermediate season; annual outdoor concentrations of air pollution were modeled at the participants’ home addresses using LUR | age, sex, education, body mass index, smoking status, physical activity, alcohol intake, neighborhood level | KORA, immunonephelometry on a BN II analyzer; HNR, a Clauss method using an automated BCS-Analyzer; FINRISK, the Clauss method using IL Test Fibrinogen-C kit and ACL300R equipment; "60-year-olds" a functional spectrophotometric test |  |
| Lee 2018 | low | **N/A** | probably low | low | low | low |
|  | from a cohort of 84,914 subjects who underwent health check-ups at the Samsung Medical Center, non-smokers or ex-smokers, >3 hospital visits, residents of one of the 25 districts of Seoul |  | daily representative district-specific concentrations:24-  hour mean values for PM_10_ | age, sex, BMI, morbidity, the proportion of green space, ambient temperature and relative humidity | a clotting assay with STA-Fibrinogen and STA-Owren Koller Buffer | yes |
| Su 2017 | low | **N/A** | probably low | low | low | low |
|  | from a cohort study on work and environment-related cardiovascular diseases as the control subjects of acute CHD in NTUH (35–65 years old) |  | LUR models were used to estimate annual average air pollution concentrations at the participants’ home addresses | age, sex, BMI, systolic BP, LDL-C, lipid-lowering treatment, diabetes mellitus, ever smoking history and hs-CRP | clotting method of Clauss and STA-Fibrinogen Kits | yes |
| Viehmann 2015 | low | **N/A** | probably low | low | low | low |
|  | from prospective cardiovascular cohort study (The German Heinz Nixdorf Recall Study) |  | EURAD chemistry transport and dispersion model, at participant’s home address | age, smoking status, ETS, economic activity, physical activity, BMI, alcohol intake, diabetes, cardiovascular disease, statin intake, season, temperature | an automated BCS-analyser | yes |
| Puett 2019 | low | low | probably low | low | low | low |
|  | from five of six original sites in the SEARCH for Diabetes in Youth Study (SEARCH), a large  multicenter observational study | based on verification of a physician diagnosis of diabetes. | spatio-temporal models to provide estimates of 24-hour average PM2.5 mass | site, age, gender, residential census tract percent below  poverty, physical activity, sedentary behavior and smoking | IL-6: a monoclonal antibody-based, high sensitive solid-phase  ELISA method;  fibrinogen: a nephelometer  autoanalyzer | yes |
| Deng 2020 | low | low | low | probably high | low | low |
|  | patients from the clinical laboratory of Changsha Central Hospital which is the biggest general hospital | patients from the clinical laboratory of Changsha Central Hospital | 11 fixed-site station located in the center of our study area | temperature, relative humidity | based on biuret colorimetry | yes |
| Zhang 2020 | probably low | **N/A** | probably low | low | low | low |
|  | college students without chronic diseases and did not used any medication or dietary supplements |  | Supersite located on the rooftop of a 15 m-high building, and approximately 9 km away from the campus | gender, age, BMI, and whether suffering an infectious disease | a commercial human cytokine/chemokine kit | yes |

Abbreviations: N/A= not applicable, AQS=Air Quality System, COPD=chronic obstructive pulmonary disease, ELISAs=enzyme-linked immunosorbent assays, hr=hour, OGTT=oral glucose tolerance test, MPO=Myeloperoxidase, GSTM1=Glutathione S-transferase M1, HbA1c=Glycosylated hemoglobin A1c, BCS=Behring Coagulation System, [tumor](#/javascript:;) [necrosis](#/javascript:;) [factor](#/javascript:;)-α=TNF-α, TEOM=tapered element oscillating microbalance, NHANES III=The Third National Health and Nutrition Examination Survey, AIRS=the Aerometric Information Retrieval System, USC=University of Southern California, NHLBI=National Heart, Lung and Blood Institute, UNC=University of North Carolina, HI=Harvard impactors, NAS=The Normative Aging Study, NTUH=National Taiwan University Hospital, HDL=high-density lipoprotein, LDL=low-density lipoprotein, MI=myocardial infarction, hs-CRP=high-sensitivity C-reactive protein, LDL-C=low-density lipoprotein cholesterol, BP=blood pressure, LUR=land-use regression, CHD=coronary heart disease, NTUH=National Taiwan University Hospital, EURAD=European Air Pollution Dispersion; BMI=body mass index, ETS= Environmental tobacco smoke





**Figure S1 Filled funnel plots of “trim-and-fill” analyses (A) short-term exposure to PM_2.5_ and IL-6 (B) long-term exposure to PM_2.5_ and fibrinogen (s.e.: standard error)**

**PRISMA Checklist**

| **#** | **Item** | **Guidance** | **On page #** | **Author Comments** |
| --- | --- | --- | --- | --- |
| **Title** | | | | |
| 1 | Title | Identify the report as a systematic review, or systematic review and meta-analysis, as appropriate. | 1 | The title identifies the report both as a systematic review and meta-analysis. |
| **Abstract** | | | | |
| 2 | Structured summary | Provide a structured summary including, as applicable:   - Background; - Objectives; - Data sources; - Study eligibility criteria, participants, and interventions; - Study appraisal and synthesis methods; - Results; - Limitations; conclusions and implications of key findings; - Systematic review registration number. | 2 | A structured abstract is provided based on the PRISMA guidelines, as:  -Background  -Objective (study criteria)  -Methods (data sources + study appraisal and synthesis methods)  -Results  -Discussion (conclusions + key findings + [future](../../../../Program%20Files%20(x86)/Youdao/Dict/8.8.0.0/resultui/html/index.html#/javascript:;) [research](../../../../Program%20Files%20(x86)/Youdao/Dict/8.8.0.0/resultui/html/index.html#/javascript:;) [direction](../../../../Program%20Files%20(x86)/Youdao/Dict/8.8.0.0/resultui/html/index.html#/javascript:;)); |
| **Introduction** | | | | |
| 3 | Rationale | Describe the rationale for the review in the context of what is already known. | 4-5 | Inflammation and coagulation cascade are considered as potential mechanisms of ambient particulate matter exposure induced adverse cardiovascular events. TNF-α, IL-6, IL-8, and fibrinogen are arguably the four most commonly assayed markers to reflect the associations of ambient particulate matter with inflammation and blood coagulation. However, the effect of PM_2.5_ and PM_10_ on the above markers are inconsistent in epidemiological studies. This review summarized and quantitatively analyzed the existed studies and promoted future research in this field. This is described in the ‘1.Introduction’. |
| 4 | Objectives | Provide an explicit Population-Intervention-Comparator-Outcome-Study Design (PICOS) or Population-Exposure-Comparator-Outcome-Study Design (PECOS) statement as appropriate, detailing the following in relation to the research questions being asked:   - Participants - Interventions / Exposures (as appropriate) - Comparisons - Outcomes - Study design | 6 | Our PECO statement includes  -Participants: humans (general population and patient), exclude pregnant women.  -Exposure: short-term and long-term exposure to PM_2.5_ and PM_10_  -Comparison: not applicable.  -Outcome: inflammation and blood coagulation markers (TNF-α, IL-6, IL-8, fibrinogen) level change  -Study design: epidemiological studies. This is described in the ‘2.2 Inclusion and exclusion criteria’ and Table S1. |
| **Methods** | | | | |
| 5 | Protocol and registration | Indicate if a review protocol exists, if and where it can be accessed (e.g. web address), and registration information including registration number (if available). |  |  |
| 6 | Eligibility criteria | Specify study characteristics (e.g. PICOS/PECOS, length of exposure) and report characteristics (e.g. years considered, language, publication status) used as criteria for eligibility, giving rationale. | 6 | The eligibility criteria were devised based on the review’s research question which was explained in the ‘introduction’. The criteria are given in the “2.2 Inclusion and exclusion criteria”. |
| 7 | Information sources | Describe all information sources (e.g. databases with dates of coverage, contact with study authors to identify additional studies) in the search, and date last searched. | 5-6 | We searched three English databases (PubMed, Web of Science, Embase) and two Chinese databases (Wang-Fang, China National Knowledge Infrastructure). References of included studies were searched to find more relevant studies. This is described in the “2.1 Search methods” and “2.3 Study selection” |
| 8 | Search | Present full electronic search strategy for at least one database, including any limits used, such that it could be repeated. | 5-6 | We searched databases using keywords that describe PM and markers and MESH terms. A full description is provided in “2.1 Search methods” and supplemental material. |
| 9 | Study selection | State the process for selecting studies (i.e., screening, eligibility, included in systematic review, and, if applicable, included in the meta-analysis). | 6-7 | “2.2 Inclusion and exclusion criteria” and “2.3 Study selection” describes the studies selection for the systematic review. In the review, we included studies met inclusion criteria. Meta-analyses were conducted only when four or more eligible studies examined the association. |
| 10 | Data collection process | Describe method of data extraction from reports (e.g., piloted forms, independently, in duplicate) and any processes for obtaining and confirming data from investigators. | 7 | Described under “2.4 Data extraction and synthesis” |
| 11 | Data items | List and define all variables for which data were sought (e.g., PICOS/PECOS, funding sources) and any assumptions and simplifications made. | 7 | Described under “2.4 Data extraction and synthesis” |
| 12 | Risk of bias in individual studies | Describe methods used for assessing risk of bias of individual studies (including specification of whether this was done at the study or outcome level), and how this information is to be used in any data synthesis. | 7 | Risk of bias is evaluated based on Office of Health Assessment and Translation (OHAT) tool. Described in “2.5 Risk of bias evaluation” |
| 13 | Summary measures | State the principal summary measures (e.g., risk ratio, difference in means). | 7 | The association between PM_2.5_ and PM_10_ and markers were expressed as percent change in markers per 10 ug/m^3^ in PM_2.5_ and PM_10_ concentration. (“2.4 Data extraction and synthesis”) |
| 14 | Synthesis of results | Describe the methods of handling data and combining results of studies, if done, including measures of consistency (e.g., I^2^) for each meta-analysis. | 8 | “2.6.1 Meta-analysis” provides a full description. Briefly, Meta-analysis was applied with a random-effect model. I^2^ index was used to estimate the heterogeneity across studies. |
| 15 | Risk of bias across studies | Specify any assessment of risk of bias that may affect the cumulative evidence (e.g., publication bias, selective reporting within studies). | 9 | Publication bias was assessed. We also performed “trim-and- fill” analysis to adjust for publication bias. Described in “2.6.3 Meta-regression, sensitivity analyses, and publication bias”. |
| 16 | Additional analyses | Describe methods of additional analyses (e.g., sensitivity or subgroup analyses, meta-regression), if done, indicating which were pre-specified. | 8-9 | Additional analyses are described in “2.6 Statistical analysis” section and includes:  1) 2.6.2 Subgroup analyses  2) 2.6.3 Meta-regression, sensitivity analyses, and publication bias |
| **Results** | | | | |
| 17 | Study selection | Give numbers of studies screened, assessed for eligibility, and included in the review, with reasons for exclusions at each stage, illustrated with a PRISMA flow diagram. | 9-10 | “3.1 Study characteristics”, including PRISMA flow diagram (Figure 1) |
| 18 | Study characteristics | For each study, present in a summary table the characteristics for which data were extracted (e.g., study size, PICOS/PECOS, follow-up period) and provide the citations. | 7-16 in supplementary material | The characteristics of the included studies are summarized in Table S3. We included the table in the supplementary material because of its length. |
| 19 | Risk of bias within studies | Present data on risk of bias of each study and, if available, any outcome level assessment (see item 12). | 10, 43, 17-37 in supplementary material | Described in”3.2 Risk of bias evaluation”, Figure 2 and Table S4 |
| 20 | Results of individual studies | For all outcomes considered (benefits or harms), present, for each study: (a) simple summary data for each intervention group (b) effect estimates and confidence intervals, ideally with a forest plot (unless such a plot would be misleading) | 10-12 | The meta-analyses results described in “3.3 Associations between PM_2.5_ and markers”, “3.4 Associations between PM_10_ and markers”. |
| 21 | Synthesis of results | Present results of each meta-analysis done, including confidence intervals and measures of consistency. | 44 | Presented in Figure 3. |
| 22 | Risk of bias across studies | Present results of any assessment of risk of bias across studies (see Item 15). | 40, 47 | Funnel plots, Begg’s test and Egger’s test are presented in Figure 6 and Table 3 |
| 23 | Additional analysis | Give results of additional analyses, if done (e.g., sensitivity or subgroup analyses, meta-regression [see Item 16]). | 10-13 | Additional analyses are described in “3.3 Associations between PM_2.5_ and markers”, “3.4 Associations between PM_10_ and markers”, “3.5 Meta-regression analysis, sensitivity analyses and publication bias” section and includes:  1) subgroup analysis  2) meta-regression analysis  3) sensitivity analyses |
| **Discussion** | | | | |
| 24 | Summary of evidence | Summarize the main findings including the strength of evidence for each main outcome; consider their relevance to key groups (e.g., researchers, users, and policy makers). | 13-16 | The main findings are discussed in “4. Discussion” session |
| 25 | Limitations | Discuss limitations at study and outcome level (e.g., risk of bias), and at review-level (e.g., incomplete retrieval of identified research, reporting bias). | 16 | The limitations of the studies are discussed as part of the Discussion section. Limitations of our review are discussed at the last paragraph of the section. |
| 26 | Conclusions | Provide a general interpretation of the results in the context of other evidence, and implications for future research. | 16-17 | Described under “ 5. Conclusion” |
| **Funding** | | | | |
| 27 | Funding | Describe sources of funding for the systematic review and other support (e.g., supply of data); role of funders for the systematic review. | 17 | The funding is described in the “Funding”. |
